# Co-expression of a cyclizing asparaginyl endopeptidase enables efficient production of cyclic peptides *in planta*

**DOI:** 10.1093/jxb/erx422

**Published:** 2017-12-22

**Authors:** Simon Poon, Karen S Harris, Mark A Jackson, Owen C McCorkelle, Edward K Gilding, Thomas Durek, Nicole L van der Weerden, David J Craik, Marilyn A Anderson

**Affiliations:** 1 Department of Biochemistry and Genetics, La Trobe Institute for Molecular Science, La Trobe University, Melbourne, Victoria, Australia; 2 Division of Chemistry and Structural Biology, Institute for Molecular Bioscience, The University of Queensland, Brisbane, Queensland, Australia

**Keywords:** Asparaginyl endopeptidase, cyclic peptide, cyclotide, kalata B1, *Nicotiana benthamiana*, plant-made pharmaceutical, transient expression, SFTI

## Abstract

Cyclotides are ultra-stable, backbone-cyclized plant defence peptides that have attracted considerable interest in the pharmaceutical industry. This is due to their range of native bioactivities as well as their ability to stabilize other bioactive peptides within their framework. However, a hindrance to their widespread application is the lack of scalable, cost-effective production strategies. Plant-based production is an attractive, benign option since all biosynthetic steps are performed *in planta*. Nonetheless, cyclization in non-cyclotide-producing plants is poor. Here, we show that cyclic peptides can be produced efficiently in *Nicotiana benthamiana*, one of the leading plant-based protein production platforms, by co-expressing cyclotide precursors with asparaginyl endopeptidases that catalyse peptide backbone cyclization. This approach was successful in a range of other plants (tobacco, bush bean, lettuce, and canola), either transiently or stably expressed, and was applicable to both native and engineered cyclic peptides. We also describe the use of the transgenic system to rapidly identify new asparaginyl endopeptidase cyclases and interrogate their substrate sequence requirements. Our results pave the way for exploiting cyclotides for pest protection in transgenic crops as well as large-scale production of cyclic peptide pharmaceuticals in plants.

## Teaser

Backbone-cyclized peptides, which have applications in the pharmaceutical and agricultural industries, can be made efficiently in plants by co-expressing them with a cyclizing enzyme.

## Introduction

Cyclotides are plant defence peptides that are exceptionally stable. They have a head-to-tail cyclic peptide backbone of around 30 amino acid residues and together with three interlocking disulfide bonds form a cyclic cystine knot structure. Cyclotides are attractive candidates for application within the pharmaceutical and agricultural industries both directly, by harnessing their intrinsic bioactivities, and indirectly by presenting foreign bioactive peptides within their stable framework (Craik *et al.*, 1999; Göransson *et al.*, 2012; Poth *et al.*, 2013; Weidmann and Craik, 2016).

Despite their complex structure, cyclotides can be produced synthetically, and numerous studies describe the synthesis of a range of native as well as engineered cyclic peptides (Daly *et al.*, 1999; Gunasekera *et al.*, 2006). These studies are generally based on solid-phase peptide synthesis and involve the use of expensive reagents and the generation of large amounts of waste. On an industrial scale, these factors could have a significant impact on the cost and environmental impact of production. Alternatively, there are various biochemical approaches, both *in vitro* and *in vivo*, for cyclizing peptides generated by standard recombinant techniques (Aboye and Camarero, 2012). These are promising for the development of cyclic peptide libraries that can be screened for biological activity and for the rapid elucidation of structure–function relationships.

Plant-based production of therapeutic proteins, although still in its infancy, has developed rapidly over the past two decades. This evolution has been driven by advantages of production speed, cost, and safety over traditional microbial and mammalian cell culture systems (Yao *et al.*, 2015). Given that cyclotides can accumulate to high levels in their native plant hosts (as high as 2 mg g^−1^ of plant material; Gran, 1970), plant-based production could be the most efficient approach for large-scale manufacture of cyclic peptides for therapeutic and other uses. Furthermore, heterologous production in plants is crucial for exploiting the potential pest control applications of cyclotides in transgenic crops. The plant species for which cyclotides have been most studied, such as *Oldenlandia affinis*, *Clitoria ternatea*, and *Viola* species, are seldom used in plant biotechnology and their amenability to gene transfer methods is not established. Regardless, large-scale cyclic peptide production would be best suited to plants that lack a background of native cyclotides and this is the case for species already established as protein production platforms such as tobacco, potato, lettuce, duckweed, and maize. However, when cyclotide genes are transferred to plants that are not native cyclotide-producers, cyclization efficiency is poor and acyclic misprocessed versions of the cyclotides predominate (Gillon *et al.*, 2008; Conlan *et al.*, 2012).

**Fig. 1.**
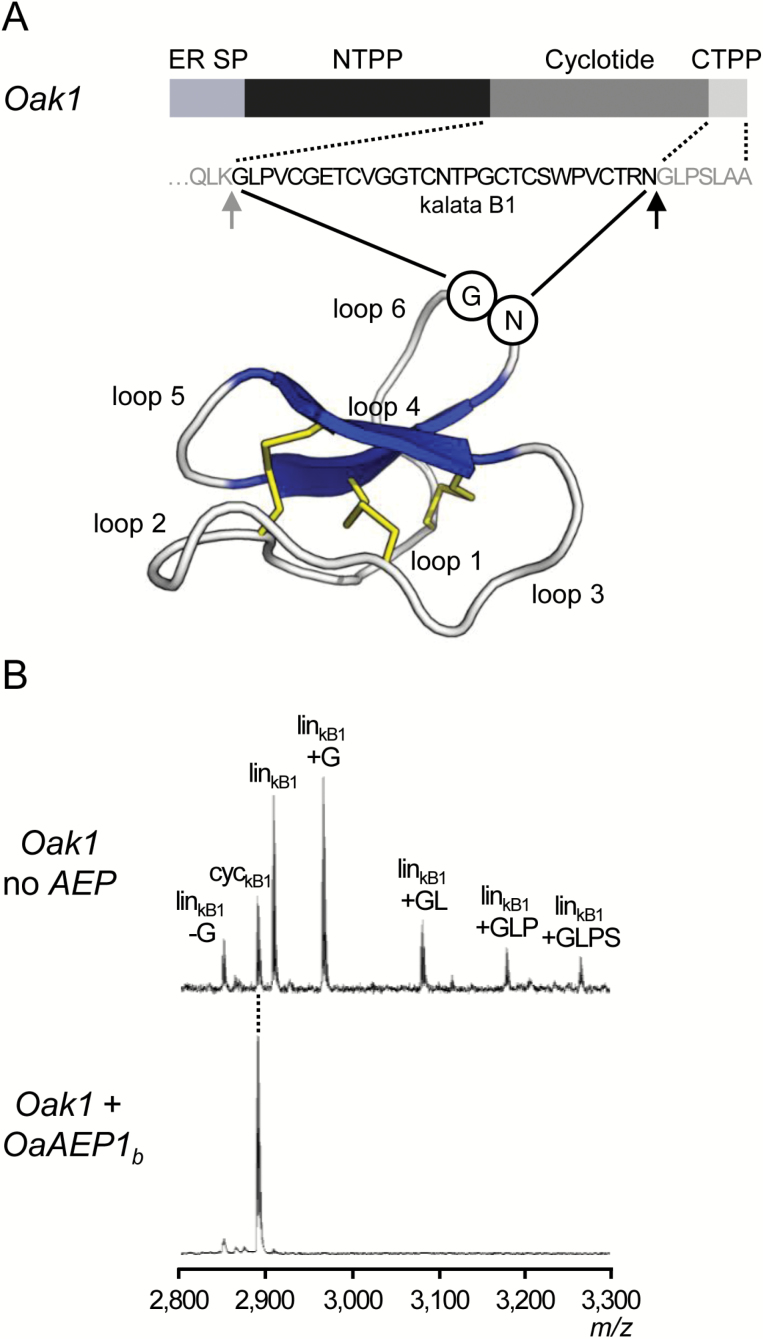
Cyclotide structure and production in *N. benthamiana*. (A) The prototypic cyclotide, kalata B1, is encoded by *Oak1*, which is composed of an ER signal peptide (ER SP), an N-terminal propeptide (NTPP), the mature cyclotide domain and a C-terminal propeptide (CTPP). The precursor protein is proteolytically processed to mature kalata B1 with the final steps involving cleavage at the NTPP (grey arrow) followed by cleavage at the CTPP (black arrow) and subsequent cyclization by peptide bond formation between the first glycine residue and the last asparagine residue of the mature cyclotide domain. The cyclic protein backbone and the knotted arrangement of three disulfide bonds (yellow) impart exceptional stability whilst the loop regions contribute native bioactivities or can be replaced with other bioactive peptides. The ribbon structure is based on PDB code 1NB1. (B) Representative MALDI-TOF mass spectra of peptides produced by transient expression of *Oak1* alone or co-expressed with *OaAEP1*_*b*_ in *N. benthamiana*. The production of predominantly linear peptides when *Oak1* is expressed alone in *N. benthamiana* has been reported previously (Gillon *et al.*, 2008; Conlan *et al.*, 2012). The position of cyclic kB1 is indicated with a dotted line; cyc, cyclic product; lin, linear product.

The maturation of cyclotide precursors involves successive enzymatic removal of the ER signal sequence and N-terminal propeptide (NTPP) followed by a transpeptidation reaction in which a C-terminal propeptide (CTPP) is released and the N- and C-termini of the cyclotide domain are joined (Gillon *et al.*, 2008; Harris *et al.*, 2015; Shafee *et al.*, 2015; Fig. 1A). This final reaction is catalysed by members of the asparaginyl endopeptidase (AEP) family of enzymes (EC 3.4.22.34; Nguyen *et al.*, 2014; Harris *et al.*, 2015). Although AEPs more commonly act as proteases, a subset have evolved a ligase function in cyclotide-producing plants (Shafee *et al.*, 2015). Plants that lack the appropriate AEP isoform required for backbone cyclization are therefore intrinsically ill-suited for recombinant cyclotide production (Gillon *et al.*, 2008).

Here we show that co-expression of a cyclizing AEP with a cyclotide gene significantly improves cyclization efficiency in non-cyclotide producing plants, resulting in a much higher proportion of cyclic product than with the cyclotide gene alone. This approach was successful in a range of food and non-food plants, and is applicable to both native and engineered cyclotides as well as the trypsin inhibitor class of cyclic peptides.

## Materials and methods

### Production of gene constructs

DNA encoding either AEPs or substrates was cloned into a vector containing the cauliflower mosaic virus 35S promoter and terminator sequences (Tabe *et al.*, 1995) before transfer into the binary vector pBIN19 (Bevan, 1984). The pBIN19 expression vectors were then transformed into *Agrobacterium tumefaciens* (strain LBA4404) by electroporation. All gene constructs were made using a variety of standard molecular biology techniques such as cDNA cloning, PCR, restriction site cloning and Gibson assembly; some sequences were also generated by gene synthesis (GenScript). The following sequences used in this study have GenBank accession numbers: *Oak1* (AF393825), *OaAEP1* (KR259377 with substitutions 9A>G and 1112A>T to produce *OaAEP1*_*b*_), *OaAEP2* (KR259378 with a single substitution 14C>T), *OaAEP3* (KR259379), *CtAEP1* (KF918345), *CtAEP2* (KR912009), *CtAEP6* (KY640209), *Oak4* (AF393828), and *Cter M* (JF501210). Other sequences are listed in Supplementary Figs S8A and S10F at *JXB* online. For co-expression of the p19 suppressor, the pEAQexpress-GFP-HT construct was used whilst *Oak1* was also expressed with pEAQ-HT-DEST1 (Sainsbury *et al.*, 2009).

### Transient expression in plants

*Agrobacterium* cells were spread as a bacterial lawn on agar plates containing yeast mannitol medium supplemented with kanamycin (50 μg ml^−1^) and streptomycin (100 μg ml^−1^). The cells were grown in the dark at 30 °C for 3 d. The lawn of bacteria was harvested and resuspended to an OD_600_ of 1.0 in infiltration buffer (10 mM MgCl_2_ and 10 μM acetosyringone). The resuspended bacteria were incubated at room temperature for 2–4 h. *Nicotiana benthamiana* plants were grown from seed in either a glasshouse or a growth cabinet at 25 °C for 4–6 weeks. In the latter, there was a 16 h/8 h day/night cycle. Leaves were infiltrated with the resuspended *Agrobacterium* (or 1:1 mixtures of the relevant *Agrobacterium* suspensions for co-expression) by gently pressing a 1 ml syringe (without a needle) against the underside of the leaf. The infiltrated area of the leaf was outlined with a permanent marker. Plants were grown for a further 4 d before the infiltrated areas were excised. Other plant tissues used were bush bean cotyledons (*Phaseolus vulgaris* cv. Royal Burgundy), grown for 10 d, and lettuce leaves (*Lactuca sativa* cv. Green Cos), grown for approximately 4 weeks. Infiltrated leaf segments were weighed, placed in microfuge tubes with a ball bearing and ground to a fine powder in liquid nitrogen using a mixer mill (30 s^−1^, 2 × 15 s). Proteins were extracted in aqueous 50% (v/v) acetonitrile–0.1% (v/v) trifluoroacetic acid (TFA) using 1 μl mg^−1^ wet weight together with 5 mg of insoluble poly(vinylpolypyrrolidone). Samples were centrifuged and the supernatants were then analysed by matrix-assisted laser desorption/ionization time of flight mass spectrometry (MALDI-TOF MS). Alternatively, whole *N. benthamiana* plants were vacuum infiltrated by submerging them in 2 litres *Agrobacterium* suspensions in a desiccation chamber. The *Agrobacterium* had been grown in liquid LB medium supplemented with antibiotics for 2 d (200 rpm, 30 °C), centrifuged and resuspended in infiltration buffer to an OD_600_ of 0.5. A vacuum was applied to 100 mbar and then slowly released; this process was repeated. The plants were then grown for a further 6 d. The total leaf tissue from each plant was weighed, lyophilized, weighed again and then ground to a fine powder using ball bearings in a tissue homogenizer (Geno/Grinder 2010) (1500 rpm, 3 min). Proteins were extracted with 50% acetonitrile–0.1% TFA using 20 ml g^−1^ dry weight.

### Stable expression in plants

*N. tabacum* and *N. benthamiana* leaf discs were used for *Agrobacterium*-mediated plant transformations (Horsch *et al.*, 1985). A single copy, homozygous line of *N. benthamiana* transformed with *OaAEP1*_*b*_ was generated by measuring copy number/zygosity of T_1_ plants using digital droplet PCR (Głowacka *et al.*, 2016) and the single copy *NbSo* gene (GenBank accession no. AEV40816) as the reference. Stably transformed canola (*Brassica napus* cv. RI64) plants were produced essentially as described (Cardoza and Stewart, 2006); for these transformations, the NptII transcription unit in the pBIN19 binary vector was replaced by a bialaphos resistance (*bar*) selectable marker gene from the cloning vector pSAT1A-ocsAocsP-bar-ocsT (Chung *et al.*, 2005) and transgenic canola shoots were selected on phosphinothricin (glufosinate ammonium).

### Peptide purification

Kalata B1 was purified from leaf tissue harvested from 17 whole *N. benthamiana* plants vacuum infiltrated with an equal mix of *Agrobacterium* suspensions harbouring the double stack expression construct *Oak1*//*OaAEP1*_*b*_ and the p19 suppressor gene construct (pEAQexpress-GFP-HT). Peptides were extracted in aqueous 50% acetonitrile–1% formic acid using 20 ml g^−1^ dry weight and filtered to remove insoluble material. The extract was then lyophilized and the crude solid redissolved in aqueous 10% acetonitrile–1% formic acid for initial solid phase extraction using a gravity fed C18 Sep-pak column (Waters). The kB1-containing fraction was detected using MALDI-TOF MS and further purified by successive RP-HPLC using Zorbax C18 columns (21.2 × 250 mm, 7 μm, 300 Å and 9.4 × 250 mm, 5 μm, 300 Å). Purity and yield of kB1-containing fractions were determined by RP-UHPLC and gravimetrically.

### Nuclear magnetic resonance spectroscopy

Nuclear magnetic resonance (NMR) spectroscopy was used to confirm the correct fold of the isolated *in planta*-produced kB1. NMR experiments were performed on a Bruker Avance III 600 spectrometer (Bruker AXS GmbH, Karlsruhe, Germany) equipped with a cryogenically cooled probe. Approximately 1 mg of peptide (99% pure by RP-UHPLC) was dissolved in 550 μl of H_2_O/D_2_O (9:1) containing 20 µg 4,4-dimethyl-4-silapentane-1-sulfonic acid as internal standard. NMR spectra, including 1D ^1^H, as well as 2D total correlation spectroscopy (TOCSY), nuclear Overhauser effect spectroscopy (NOESY) and ^1^H-^13^C heteronuclear single quantum correlation (HSQC) spectra were recorded at 298 K and used for sequential assignment of the peaks from individual amino acids. The resultant Hα-chemical shifts provide a sensitive probe of peptide secondary and tertiary structure and were identical to literature values for kB1 isolated from *Oldenlandia affinis* (Rosengren *et al.*, 2003) (see Supplementary Fig. S3).

### Peptide analysis by MALDI-TOF MS

Leaf extracts were desalted and concentrated using C18 ZipTips (Merck Millipore) and then mixed 1:1 with α-cyano-4-hydroxycinnamic acid (5 mg ml^−1^ in 50% acetonitrile–0.1% TFA–5 mM (NH_4_)H_2_PO_4_) before they were spotted onto a ground steel sample plate and air dried. Mass analysis was performed in positive ion reflector mode on a Bruker Ultraflex III MALDI TOF/TOF mass spectrometer. Two hundred spectra at each of 10 randomly selected positions were accumulated per spot between 1000 and 4000 Da. Calibration was conducted using a mixture of peptide standards (Bruker Daltonics). Data were acquired and processed using Bruker flexAnalysis software; peaks were detected using the software’s ‘Snap’ algorithm. For relative quantification of cyclic and linear peptides, the sum of the integrated peak areas of the first three isotopic peaks corresponding to each assigned peptide was taken as 100% of the expressed peptides. The percentage of cyclic and linear peptides within the sample could then be calculated (Conlan *et al.*, 2012). Peptide assignments and relative quantification of cyclic and linear products from all transiently expressed constructs are shown in Supplementary Figs S2, S4, S5 and S8–S10.

### Absolute quantification of cyclic kB1

A MALDI-TOF MS-based method to quantify the cyclic kB1 present in *N. benthamiana* extracts was based on a previous study (Saska *et al.*, 2008) with the difference being the use of a ^15^N-labelled cyclic kB1 (Mylne and Craik, 2008) as the internal standard. Synthetic oxidized kB1 (0.078–1.25 μg ml^−1^ final concentration) was mixed with ^15^N-kB1 (0.53 μg ml^−1^ final concentration). Samples were then mixed 1:1 with α-cyano-4-hydroxycinnamic acid and analysed by MALDI-TOF MS as above. The solvent was 50% acetonitrile–0.1% TFA and all data were collected in a background of endogenous *N. benthamiana* protein extract at a final concentration of 0.45 mg ml^−1^. Peaks were detected and peak areas integrated using the ‘Centroid’ algorithm of the flexAnalysis software. The relative peak area of unlabelled to labelled kB1 was plotted against unlabelled kB1 concentration to generate a standard curve (see Supplementary Fig. S7A). For quantification of the kB1 content in unknown samples, the total protein concentration was determined by BCA assay (Pierce) and adjusted to 0.9 mg ml^−1^ using 50% acetonitrile–0.1% TFA. This was then mixed 1:1 with ^15^N-kB1 (0.53 μg ml^−1^ final concentration) and analysed in triplicate by MALDI-TOF MS as above.

## Results

### Co-expression of a cyclizing AEP with a cyclotide precursor produces a high proportion of cyclic product

**Fig. 2.**
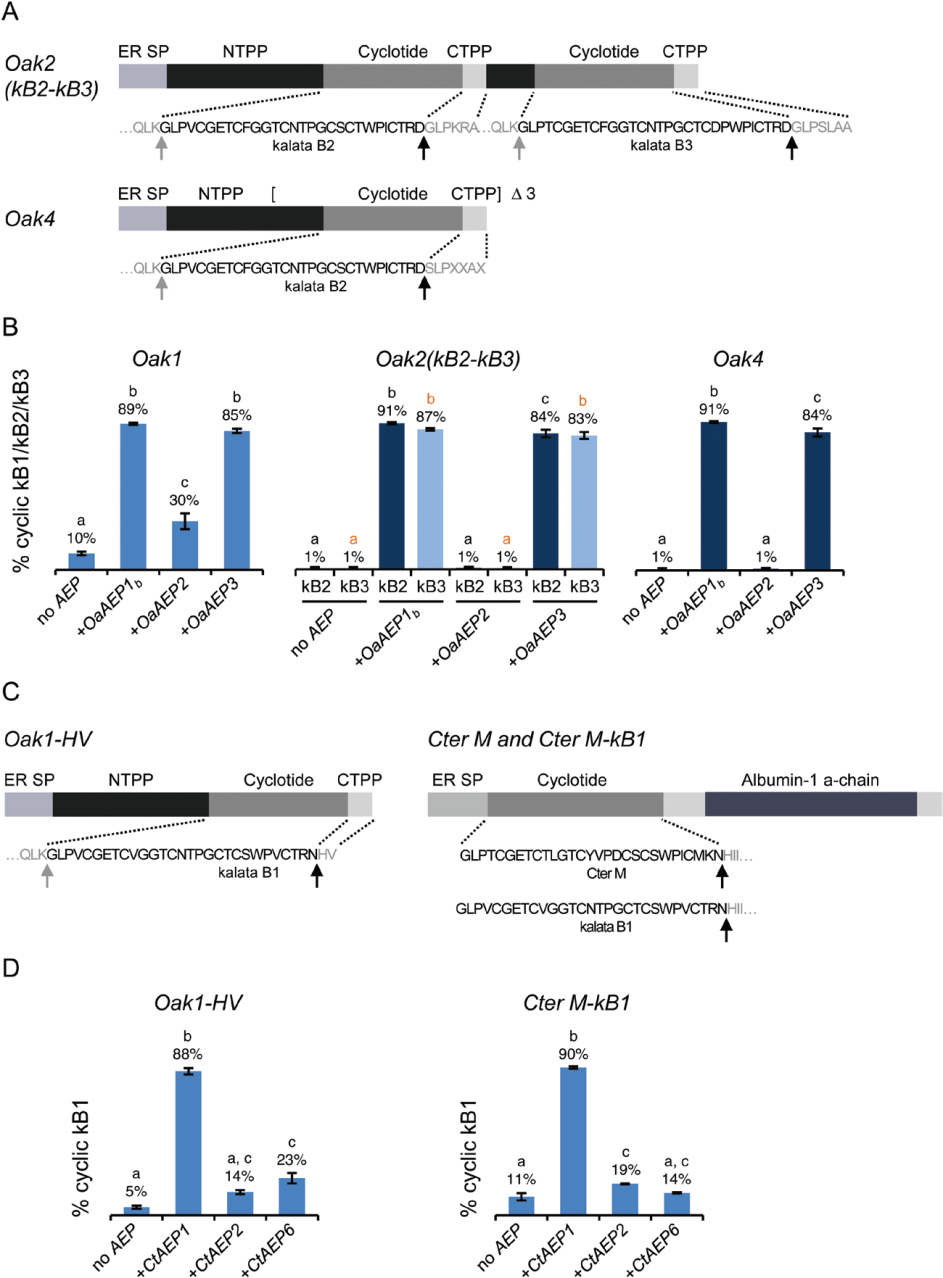
Production of kalata B1, kalata B2 and kalata B3 by transient co-expression of the cyclotide precursors with a cyclizing AEP in *N. benthamiana*. (A) *Oak2(kB2-kB3*) and *Oak4* are multidomain cyclotide sequences that present different residues from *Oak1* at the P1-P1′ positions of the C-terminal AEP processing site (*Oak1*: NG; *Oak2*: DG; *Oak4*: DS). See Fig. 1 for a description of domains and processing steps. (B) Mean percentage of cyclic kB1 (blue bars), cyclic kB2 (dark blue bars), and cyclic kB3 (light blue bars) relative to all assigned peptides±SEM based on mass spectra peak areas for *Oak1*, *Oak2(kB2-kB3*), and *Oak4* expressed alone or co-expressed with *OaAEP1*_*b*_, *OaAEP2*, or *OaAEP3*. (C) Schematic representation of substrates co-infiltrated with AEPs from *Clitoria ternatea*. In *Oak1-HV* the native CTPP sequence of *Oak1* (GLPSLAA) was replaced with HV. *Cter M* is a native cyclotide precursor from *C. ternatea* comprising the cyclotide domain embedded within an albumin precursor protein (Poth *et al.*, 2011). In *Cter M-kB1*, the native cyclotide domain was replaced with kB1 within the Cter M precursor. (D) Mean relative percentage of cyclic kB1 relative to all assigned peptides±SEM based on mass spectra peak areas for *Oak1-HV* and *Cter M-kB1* expressed alone or co-expressed with *CtAEP1*, *CtAEP2*, or *CtAEP6*. All data were derived from a minimum of three independent replicates. Different letters indicate significant differences found by Tukey’s ANOVA (*P*<0.05). Representative MALDI-TOF mass spectra, number of replicates, observed monoisotopic masses and mean relative percentages of all assigned peptides are shown in Supplementary Fig. S2.

We used transient gene expression in *Nicotiana benthamiana*, a non-cyclotide producing plant and a common platform for producing heterologous proteins, to deliver both *Oak1* (encoding the precursor to the prototypic cyclotide kalata B1 [kB1]) and a cyclizing AEP (OaAEP1_b_ from *Oldenlandia affinis* or CtAEP1 (butelase 1) from *Clitoria ternatea*) by syringe agroinfiltration (Sparkes *et al.*, 2006). In the first example, *Oak1* and the cyclizing AEP were placed under the control of the constitutive cauliflower mosaic virus 35S promoter and terminator, and transiently expressed alone or in combination. *Oak1* alone produced a suite of peptides whose masses correspond to linear oxidized kB1 along with other linear forms that lack the N-terminal Gly (−G) and/or retain successive residues from the CTPP (+G, +GL, +GLP) (Fig. 1B). Some cyclic kB1 was observed, but this constituted only a minor proportion of the total kB1 products detected. None of these peptides was detected in leaves infiltrated with empty vector or *OaAEP1*_*b*_ alone (see Supplementary Fig. S1). However, when *OaAEP1*_*b*_ was co-infiltrated with the cyclotide precursor, the relative proportion of cyclic kB1 increased substantially (Figs 1B and 2B and Supplementary Fig. S2). This increase also occurred when *CtAEP1* was co-infiltrated with *Oak1-HV*, which is *Oak1* with its CTPP changed from GLPSLAA to HV to reflect the reported specificity of CtAEP1 (Nguyen *et al.*, 2014) (Fig. 2C, D and Supplementary Fig. S2). Cyclic kB1 produced transiently in *N. benthamiana* was isolated by HPLC and the integrity of its 3D structure was confirmed by NMR (see Supplementary Fig. S3).

Kalata B2 and kalata B3, which contain different C-terminal precursor processing sites (D_29_–G_30_/S_30_ and D_30_–G_31_, respectively) from the kB1 precursor (N_29_–G_30_), were also efficiently produced when constructs containing multiple cyclotide domains were co-infiltrated with *OaAEP1*_*b*_ demonstrating that the inter-plant transfer of processing capability is not limited to the prototypic cyclotide, kB1 (Fig. 2A, B and Supplementary Fig. S2). Furthermore, a *C. ternatea* cyclotide, Cter M, was cyclized by CtAEP1, though not by OaAEP1_b_, when presented within its native *Cter M* precursor gene (see Supplementary Fig. S4A, B). The *Cter M* precursor is atypical since the cyclotide domain is embedded within an albumin precursor that lacks the canonical NTPP sequence; in this case the ER signal sequence is directly followed by the cyclotide domain, eliminating the requirement for enzymatic removal of the NTPP (Poth *et al.*, 2011; Fig. 2C). Importantly, kB1 could also be presented within the *Cter M* albumin precursor and was efficiently cyclized *in planta* by both CtAEP1 (Fig. 2D) and OaAEP1_b_ (Supplementary Fig. S4C). These data highlight the robustness of this approach: it is applicable to multiple cyclotides presented in single and multi-domain formats as well as within typical or atypical cyclotide precursors.

The high efficiency of cyclotide maturation was independent of whether the substrate and cyclizing AEP were in separate constructs that were co-infiltrated, or in a single, double-stack construct (see Supplementary Fig. S5). In the latter configuration, the substrate and AEP remain under control of their own promoter and terminator, but are on a single T-DNA, streamlining transformations. We also produced *N. benthamiana* transgenic plants that constitutively express either *OaAEP1*_*b*_ or *OaAEP3*. Transient expression of *Oak1* alone within these AEP-expressing plants also resulted in an increased proportion of cyclic kB1 across multiple plant lines (Supplementary Fig. S6). Thus, a plant line optimally expressing a cyclizing AEP could be used for producing cyclic peptides by transient expression of the appropriate substrate.

The yield of cyclic kB1 was determined using MALDI-TOF MS (Saska *et al.*, 2008) to be 75 ± 7 μg g^−1^ dry weight (DW) of leaf tissue from *N. benthamiana* plants transiently expressing the *Oak1*//*OaAEP1*_*b*_ double stack construct. This represents at least an 8-fold increase in absolute cyclic kB1 production compared with expressing *Oak1* without *OaAEP1*_*b*_, where the cyclic kB1 yield was below the quantification limit (<8.5 μg g^−1^ DW) (see Supplementary Fig. S7B). A further increase in yield was achieved when the *Oak1*//*OaAEP1*_*b*_ double stack construct was co-expressed with the p19 suppressor of gene silencing from tomato bushy stunt virus (Scholthof, 2007) (139 ± 13 μg g^−1^ DW). As an alternative strategy for increased yield, we also expressed *Oak1* using a pEAQ vector (Sainsbury *et al.*, 2009) in a stable transgenic *N. benthamiana* line expressing *OaAEP1*_*b*_, obtaining 199 ± 21 μg g^−1^ DW compared with <10.8 μg g^−1^ DW when this construct was expressed in wild-type *N. benthamiana* (Supplementary Fig. S7B).

### Evaluation of substrate sequence requirements for cyclization in planta

Oak1 sequence requirements for the production of mature, cyclic kB1 were earlier investigated in *N. benthamiana* without co-expression of an AEP (Gillon *et al.*, 2008; Conlan *et al.*, 2012). However, this approach was limited by the low proportion of cyclic kB1 produced, suggesting that the endogenous *N. benthamiana* AEPs do not act preferentially as cyclases. Having demonstrated that co-expression of a cyclizing enzyme in *N. benthamiana* resulted in efficient cyclization of kB1, we re-examined Oak1 sequence requirements by co-expressing a series of *Oak1* variants with *OaAEP1*_*b*_ (see Supplementary Fig. S8). The minimal CTPP for efficient cyclization was Gly_30_Leu_31_, and strict requirement for Asn_29_ or Asp_29_ as the C-terminal residue was observed. Gly30Ala and Leu31Ala substitutions were also acceptable, although the proportion of cyclic product from the latter decreased from 89% to 69%.

### Screening AEPs for cyclization ability

AEPs are typically thought of as proteases and thus far only two have been identified as preferential cyclases (Shafee *et al.*, 2015). Therefore, another application of our expression system is the rapid screening of uncharacterized AEPs for cyclizing ability. To investigate their preferred function, previously uncharacterized AEPs from *O. affinis* (Harris *et al.*, 2015) and *C. ternatea* (Gilding *et al.*, 2016) were co-expressed with several different substrates (Fig. 2B, D). OaAEP3 had similar cyclization efficiency to OaAEP1_b_, producing a high proportion of cyclic kB1, kB2, and kB3, identifying it as a preferential cyclase. In contrast, OaAEP2 increased the proportion of cyclic kB1 from *Oak1* only slightly (30% cyclic product compared with 10% without AEP co-expression). Furthermore, the reduction in the proportion of linear species retaining residues from the CTPP suggests that this AEP acts predominantly as a protease, removing the CTPP without subsequent cyclization (see Supplementary Fig. S7A). OaAEP2 had no effect on the processing of kB2 or kB3 from *Oak2(kB2-kB3*) or *Oak4*, generating profiles very similar to those obtained without AEP co-expression (Supplementary Fig. S7B, C). This suggests that OaAEP2 may have a substrate preference for cleaving N–G rather than D–G or D–S.

CtAEP2 and CtAEP6 were also poor cyclases, increasing the proportion of cyclic product from *Oak1-HV* only slightly (14% and 23% cyclic product, respectively, compared with 5% without AEP co-expression and 88% for CtAEP1). Furthermore, they generated only 19% and 14% cyclic product, respectively, from the *Cter M-kB1* substrate compared with 11% without AEP co-expression and 90% for CtAEP1. Like OaAEP2, CtAEP6 eliminated the accumulation of linear processing variants carrying residues from the CTPP, suggesting efficient CTPP cleavage and a role as a protease (see Supplementary Fig. S7D, E).

### Successful cyclotide production is transferrable to crop species

Two double-stack constructs (*Oak1*//*OaAEP1*_*b*_ and *Oak1-HV*//*CtAEP1*) were transiently expressed in bush bean and lettuce and compared with constructs expressing the respective substrates alone. In each case, the presence of the cyclizing enzymes significantly increased the proportion of cyclic kB1, showing that this technology is transferrable to other plants, including edible species (see Supplementary Fig. S9). Efficient cyclic kB1 production observed in transient expression assays also extended to the stable transformation of two tobacco species (*N. tabacum* and *N. benthamiana*) and canola (Supplementary Fig. S6).

### Grafted versions of kalata B1 can be produced *in planta*

**Fig. 3.**
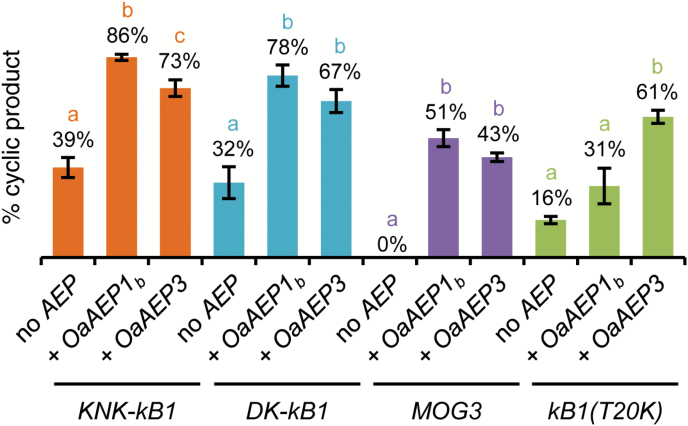
Production of grafted cyclic peptides in *N. benthamiana*. Mean relative percentage of cyclic peptide relative to all assigned peptides±SEM based on mass spectra peak areas for *Oak1-KNK-kB1* (orange bars), *Oak1-DK-kB1* (aqua bars), *Oak1-MOG3* (purple bars), and *Oak1-kB1(T20K*) (green bars) expressed alone or co-expressed with *OaAEP1*_*b*_ or *OaAEP3*. All data were derived from a minimum of three independent replicates. Different letters indicate significant differences found by Tukey’s ANOVA (*P*<0.05). Representative MALDI-TOF mass spectra, number of replicates, observed monoisotopic masses and mean relative percentages of all assigned peptides are shown in Fig. S10.

We first tested whether two kB1 variants ([W19K,P20N,V21K]kB1 (KNK-kB1) and [P20D,V21K]kB1 (DK-kB1)) engineered to explore the structural plasticity of the cyclotide framework (Clark *et al.*, 2006) could be made in *N. benthamiana*. Although the proportion of cyclic product for both molecules was higher than obtained with native kB1 when they were expressed without a cyclizing AEP (KNK-kB1, 39%; DK-kB1, 32%; native kB1, 10%), the proportion of cyclic product was significantly increased when an AEP was co-expressed (*OaAEP1*_*b*_: KNK-kB1 86%, DK-kB1 78%; *OaAEP3*: KNK-kB1 73%, DK-kB1 67%) (Fig. 3). Next, we tested the *in planta* production of two kB1 variants (MOG3 and kB1(T20K)) that have shown promising activity in a mouse model of multiple sclerosis (Wang *et al.*, 2014; Thell *et al.*, 2016). Cyclic MOG3 was not detected when the precursor was expressed without a cyclizing AEP but comprised 51% and 43% of total MOG3 peptides when co-expressed with *OaAEP1*_*b*_ or *OaAEP3*, respectively. More cyclic kB1(T20K) was also produced in *N. benthamiana* when co-expressed with a cyclizing AEP. For this molecule, *OaAEP3* produced a higher proportion of cyclic kB1(T20K) than *OaAEP1*_*b*_ with 61% cyclic peptide compared with 31% (Fig. 3).

### The cyclic peptide SFTI-1 can be produced *in planta*

To test whether this approach would also be successful for a different class of cyclic peptide, the kB1 sequence within the *Oak1* precursor was replaced with the sunflower trypsin inhibitor, SFTI-1 (Mylne *et al.*, 2011). The peptide was not detected when the precursor was expressed alone in *N. benthamiana*, but upon co-expression with *OaAEP1*_*b*_, cyclic SFTI-1 was efficiently produced (see Supplementary Fig. S10E). Successful *in planta* production was also extended to an engineered SFTI-1 variant (SFTI-FCQR) that inhibits human kallikrein-related peptidase 4, a target for prostate cancer treatment (Supplementary Fig. S10E) (Swedberg *et al.*, 2009).

## Discussion

Understanding the mechanism of cyclotide biosynthesis advanced significantly when it was shown that members of the asparaginyl endopeptidase (AEP) family of enzymes catalyse the backbone cyclization of cyclotides (Nguyen *et al.*, 2014; Harris *et al.*, 2015). When cyclotide genes are expressed without a cyclizing AEP in plants that do not natively produce cyclotides, the efficiency of cyclization is poor. Instead, endogenous enzymes, which may include AEPs that are preferential proteases with limited cyclase activity and/or carboxypeptidases that trim successive residues from the CTPP, produce predominantly linear, misprocessed versions of the cyclotide. In each of the plants tested in our study (*N. benthamiana*, *N. tabacum*, bush bean, and lettuce), none of which natively produce cyclotides, co-expression of a cyclizing AEP with a cyclotide precursor outcompeted endogenous processes that produce linear forms. Thus, the low cyclizing efficiency of plants that do not naturally produce cyclotides can be overcome.

By co-expressing previously uncharacterized AEPs with *Oak1*, we have identified OaAEP3 as another cyclizing AEP. OaAEP3 shares 79% identity with OaAEP1_b_ and both enzymes cyclized kalata B1, B2, and B3 with similar efficiency *in planta*. However, OaAEP3 was significantly better at cyclizing kB1(T20K), suggesting subtle differences in substrate specificities. It should be noted that while an AEP that produces a high proportion of cyclic product can be categorized as a cyclizing AEP, low apparent cyclization may result from other factors, such as sub-optimal substrates, low expression levels or slow kinetics such that cyclization is outcompeted by degradation by endogenous enzymes.

Previous investigations into Oak1 sequence requirements for cyclization involved the expression of *Oak1* variants in *N. benthamiana*, *N. tabacum*, or *A. thaliana* (Gillon *et al.*, 2008; Conlan *et al.*, 2012). The low cyclization efficiency for wild-type *Oak1* in these plants, however, hampered detailed substrate structure–cyclization efficiency studies. Moreover, results may also be influenced by the nature of the endogenous AEPs whose primary roles are unlikely to involve cyclization. The highly efficient cyclization of *Oak1* by *OaAEP1*_*b*_ makes our *in planta* system ideal for investigating substrate sequence requirements for cyclization. For example, in previous studies without AEP co-expression, no cyclic product was observed from the Leu31Ala variant (Conlan *et al.*, 2012) or Asn29Asp variant (Gillon *et al.*, 2008) whereas with *OaAEP1*_*b*_ co-expression, we observed 69% and 87% cyclic kB1, respectively. The reduction in the relative proportion of cyclic kB1 in the former is consistent with the slower *in vitro* processing of this precursor with recombinant purified enzyme (Harris *et al.*, 2015). The minimal CTPP for efficient cyclization (Gly_30_Leu_31_) and strict requirement for Asn_29_ or Asp_29_ as the C-terminal residue were also consistent with experiments with recombinant purified OaAEP1_b_ (Harris *et al.*, 2015). Our system therefore facilitates rapid, accurate screening of optimal precursor substrate sequences for a given AEP.

The quantification of cyclic kB1 produced in *N. benthamiana* shows that the increase in the relative proportion of cyclic kB1 produced from *Oak1* alone or co-expressed with *OaAEP1*_*b*_ (from 10% to 89%) also applies in absolute terms where the *Oak1*//*OaAEP1*_*b*_ double stack produced around 9-fold more cyclic kB1 than *Oak1* alone (see Supplementary Fig. S7B). Although the highest levels of kB1 reported here (199 μg g^−1^ DW) are around 10-fold less than present in the leaves of the native plant, *O. affinis*, (1.82 mg g^−1^ DW for plants grown in a greenhouse) (Seydel and Dörnenburg, 2006), large amounts of *N. benthamiana* leaf biomass can be grown rapidly for recombinant protein production by agroinfiltration (Chen *et al.*, 2013; Holtz *et al.*, 2015), offering a feasible option for large-scale cyclic peptide production. The reported yields of other plant-produced therapeutic proteins, such as monoclonal antibodies and vaccines (Gleba *et al.*, 2014), are generally much higher on a mass basis than what we have demonstrated with cyclic kB1. However the much smaller size of cyclotides means that on a molar basis, our current best yield is broadly comparable. Moreover, other strategies, such as the use of self-replicating plant virus-based expression vectors (Gleba *et al.*, 2014), might further increase cyclotide yield.

Cyclic peptides have also been produced *in vivo* using yeast and bacterial expression systems. In this approach, backbone cyclization is achieved by intein-mediated splicing and cyclic peptide yields of up to 180 µg l^−1^ have been reported (Jagadish *et al.*, 2013, 2015; Li *et al.*, 2016). Whilst the focus of these studies has been the production of cyclotide libraries that could be rapidly screened for a desired biological activity, this approach may also be applicable to large-scale cyclotide production. Further work could compare the feasibility of the plant and microbe systems for this purpose.

The exceptional stability of cyclotides and their reported orally delivered bioactivity from plant material, even after boiling (Gran, 1970), provides tantalising evidence that bioactive cyclotides could be introduced into edible plants and directly administered as medicines. Successful transient cyclic peptide production in *N. benthamiana* was transferrable to both bush bean and lettuce as well as stably introduced into canola. Although we have focused on production in leaf, it will be necessary to investigate other plant tissues such as seeds, fruits, and tubers to determine how broadly cyclic peptides can be produced in edible plant tissues.

The intrinsic insecticidal and nematicidal activity of some cyclotides (Jennings *et al.*, 2001; Gilding *et al.*, 2016) means that their production in transgenic crops could confer pest protection. Our evidence that cyclotides can be efficiently produced in bush bean, lettuce, tobacco and canola by co-expressing a cyclizing AEP suggests that this approach could be applied to protect agriculturally important crops. Plants that naturally produce cyclotides typically contain dozens of different cyclotides that accumulate in different tissues, suggesting that they are combinatorial mediators of defence (Gilding *et al.*, 2016). While cyclotide-producing plants are likely to express multiple AEPs, our evidence that OaAEP1_b_ cyclizes multiple cyclotides with high efficiency suggests an approach where a transgenic crop constitutively expressing a single cyclizing AEP but multiple cyclotide precursors could be protected against a broad range of pests. Alternatively, such a strategy could be used to pyramid cyclotide-based pest-protective traits, analogous to the stacking of Bt genes (Zhao *et al.*, 2003), to delay the onset of resistance.

To be broadly applicable for agricultural or pharmaceutical applications, it is important that non-native cyclotides with other, engineered activities can also be produced *in planta*. Our data confirm that the cyclization efficiency of non-native cyclotides *in planta* can be improved when a cyclizing AEP is co-expressed. The relative percentages of cyclic product for these grafted kB1s upon AEP co-expression were lower than those for native kB1 and there was a significant difference in the cyclization efficiency for kB1(T20K) between OaAEP1_b_ and OaAEP3. These grafted kB1s all have the same sequences around the AEP recognition/cleavage site as native kB1. Therefore we speculate that the grafted peptides are creating subtle conformational changes that affect the efficiency of AEP processing. Alternatively, these changes could be favourable for processing by endogenous, non-cyclizing enzymes. Determining optimal substrate–cyclizing enzyme combinations will be crucial for maximizing the proportion of cyclic product.

The successful production of a different class of cyclic peptide, the trypsin inhibitor SFTI-1 and a grafted version of this molecule, suggests that co-expression of a cyclizing AEP may find broad application for the production of a range of cyclic peptides. The absence of linear SFTI-1 products, in both the presence and the absence of the cyclizing AEP, suggests that misprocessed molecules may be rapidly degraded by endogenous enzymes, highlighting the stabilizing effect of cyclization.

In conclusion, plants can be used for the production of natural cyclotides and other cyclic peptides engineered for therapeutic use, by co-expressing target peptides with a cyclizing AEP. The establishment of agroinfiltration for heterologous protein production in *N. benthamiana* on an industrial scale (Chen *et al.*, 2013; Holtz *et al.*, 2015) means that large-scale, affordable production of cyclic peptides may be possible. Furthermore, the applicability of this technology to food crops establishes the feasibility of supplying therapeutic cyclotides directly from plant sources or exploiting the potential of cyclotides to enhance pest resistance in transgenic crops. Finally, this approach can also be used to identify and characterize AEPs with cyclizing ability and to probe substrate sequence requirements for cyclization *in planta*.

## Supplementary data

Supplementary data are available at *JXB* online.

Fig. S1. Representative MALDI-TOF MS spectra of *Oak1* transiently expressed in *N. benthamiana* compared with transient expression of *OaAEP1*_*b*_ or an empty vector.

Fig. S2. Production of kalata B1, kalata B2 and kalata B3 by transient co-expression of the cyclotide precursors with a cyclizing AEP in *N. benthamiana*.

Fig. S3. NMR analysis of kB1 isolated from *N. benthamiana* transiently expressing *Oak1*//*OaAEP1*_*b*_.

Fig. S4. Transient expression of cyclotides in the *Cter M* precursor.

Fig. S5. Transient expression of double stack constructs.

Fig. S6. Cyclotide production in stable transformants.

Fig. S7. MALDI-TOF MS quantification of cyclic kalata B1 produced in *N. benthamiana*.

Fig. S8. Transient co-expression of *Oak1* variants with *OaAEP1*_*b*_ in *N. benthamiana*.

Fig. S9. Cyclotide production in bush bean and lettuce.

Fig. S10. Production of grafted cyclic peptides in *N. benthamiana*.

Supplementary Figures S1-S10

## Abbreviations:

AEP: asparaginyl endopeptidase
CTPP: C-terminal propeptide
kB1: kalata B1
NTPP: N-terminal propeptide
SFTI: sunflower trypsin inhibitor.
