# Supplementary material for: Co-expression of a cyclizing asparaginyl endopeptidase enables efficient production of cyclic peptides in planta
Source: J Exp Bot. 2017 Dec 22;69(3):633–41. doi: 10.1093/jxb/erx422 (PMC5853369; doi:10.1093/jxb/erx422)
Supplement: Supplementary Figures S1-S10 [file erx422_suppl_supplementary_figures_s1-s10.pdf]

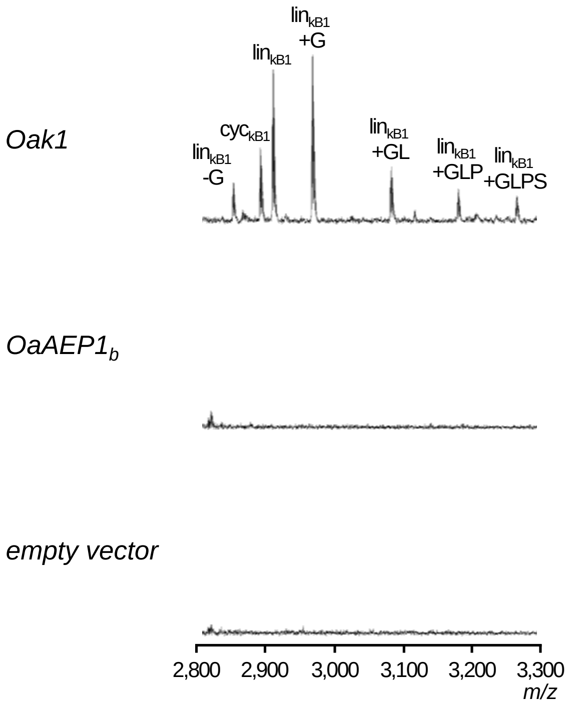

Fig. S1. Representative MALDI-TOF MS spectra of *Oak1* transiently expressed in *N. benthamiana* compared to transient expression of *OaAEP1*$_b$ or an empty vector. The scales for all spectra are the same; cyc, cyclic product; lin, linear product.

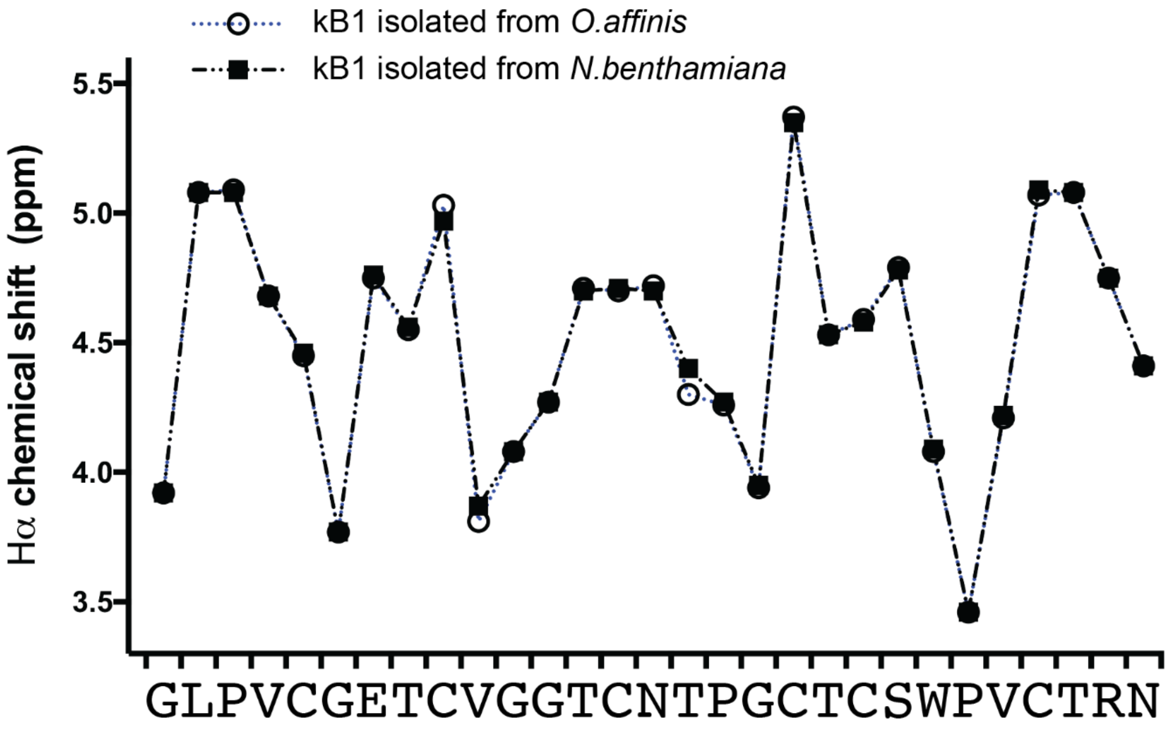

Fig. S2. NMR analysis of kB1 isolated from *N. benthamiana* transiently expressing *Oak1*//*OaAEP1*$_b$. The Hα chemical shift of each residue provides a sensitive probe of peptide secondary and tertiary structure. The obtained values were compared to kB1 isolated from *Oldenlandia affinis* (the native kB1-producing plant) (Rosengren *et al.,* 2003) and indicate that the molecules are identical. Minor deviations of ≤ 0.1 ppm are due to slight differences in the pH of the NMR samples.

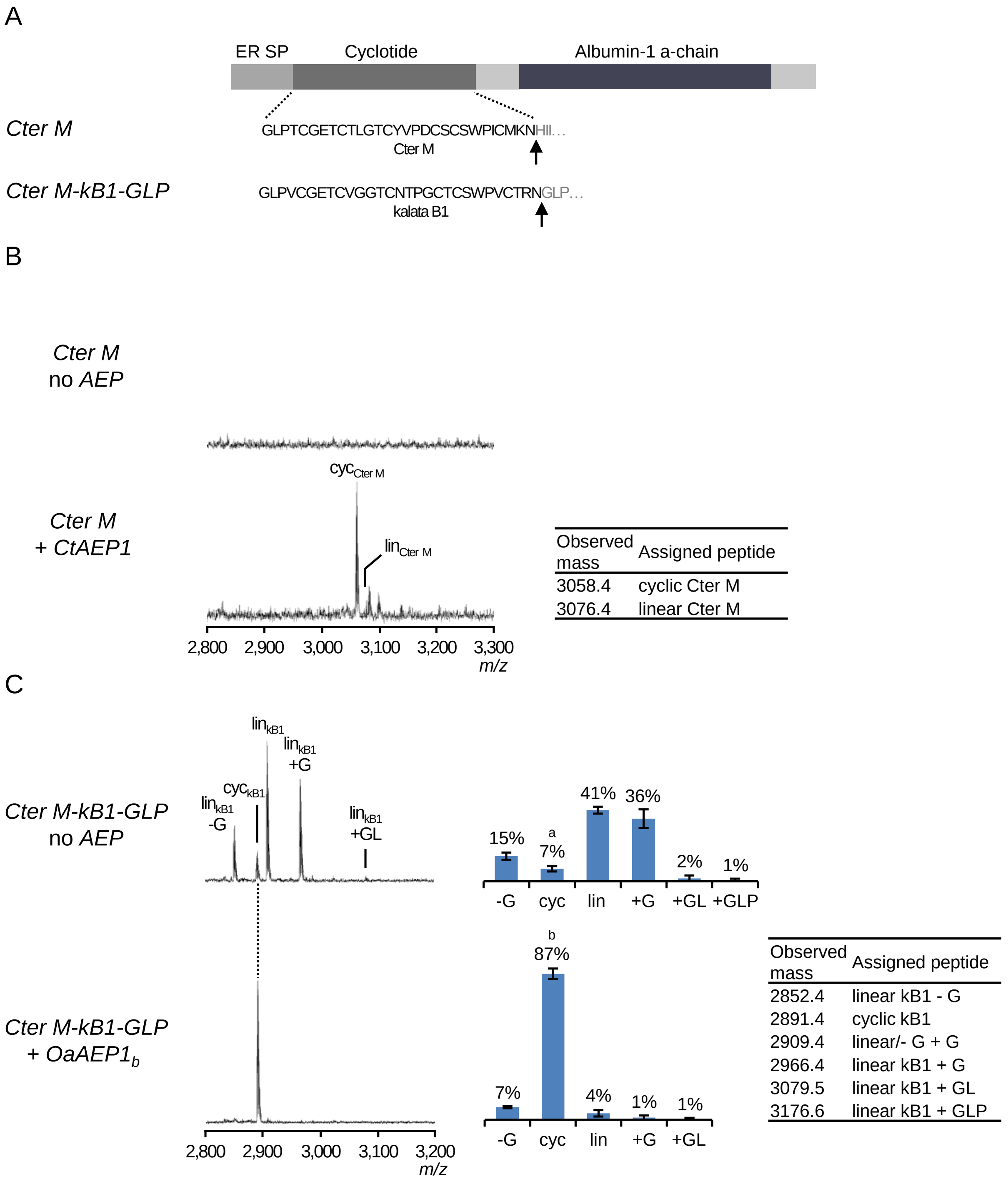

Fig. S3. Transient expression of cyclotides in the *Cter M* precursor. (A) Domain structure of Cter M. For Cter M-kB1-GLP, the Cter M cyclotide domain and the HII tripeptide were replaced with kalata B1 and GLP. The arrows indicate the AEP processing sites. Representative MALDI-TOF MS spectra of (B) *Cter M* and (C) *Cter M-kB1-GLP* transiently expressed with or without *CtAEP1* and *OaAEP1*$_b$, respectively in *N. benthamiana*. The scales for the *Cter M* ± *CtAEP1* spectra are the same. For *Cter M-kB1-GLP* ± *OaAEP1*$_b$, the position of cyclic kB1 is indicated with a dotted line; cyc, cyclic product; lin, linear product. The bar graphs show the mean relative percentages of cyclic and linear products relative to all assigned peptides ± SEM based on mass spectra peak areas (n=3, except *Cter M-kB1-GLP* + *OaAEP1*$_b$ (n=5)). Different letters indicate a significant difference found by Tukey's ANOVA ($p<0.05$). The tables show observed monoisotopic masses (Da; $[M+H]^+$) and assigned peptides.

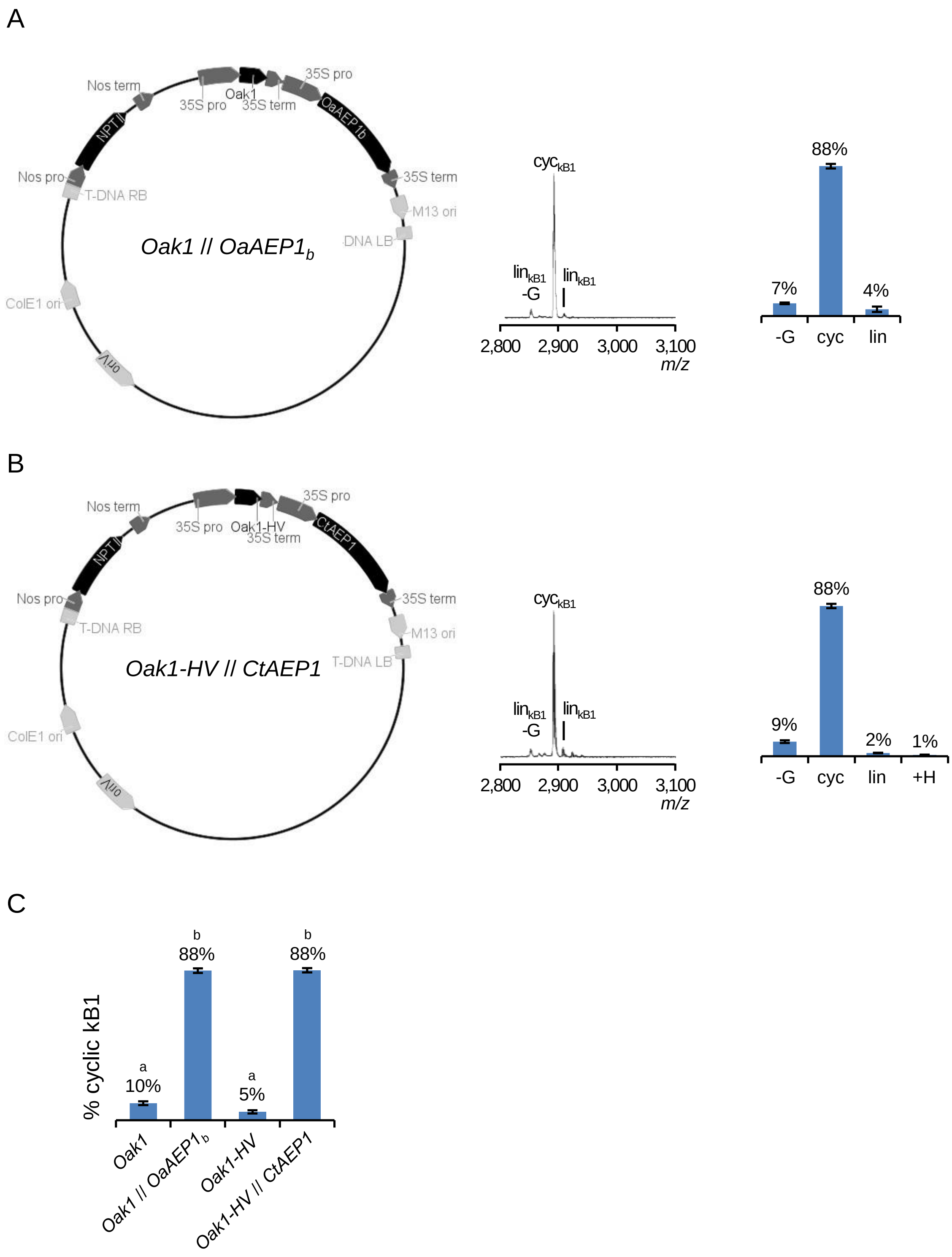

Fig. S4. Transient expression of double stack constructs. Plasmid maps for the (A) *Oak1//OaAEP1$_b$* and (B) *Oak1-HV//CtAEP1* double stack constructs, representative MALDI-TOF MS spectra of these constructs transiently expressed in *N. benthamiana* and mean percentages of cyclic and linear products relative to all assigned peptides ± SEM based on mass spectra peak areas (n=3 and 4 for (a) and (b), respectively); cyc, cyclic product; lin, linear product. (c) Mean percentage of cyclic kB1 relative to all assigned peptides for each double stack construct compared to the respective substrates alone; different letters indicate significant differences found by Tukey's ANOVA (p<0.05).

A

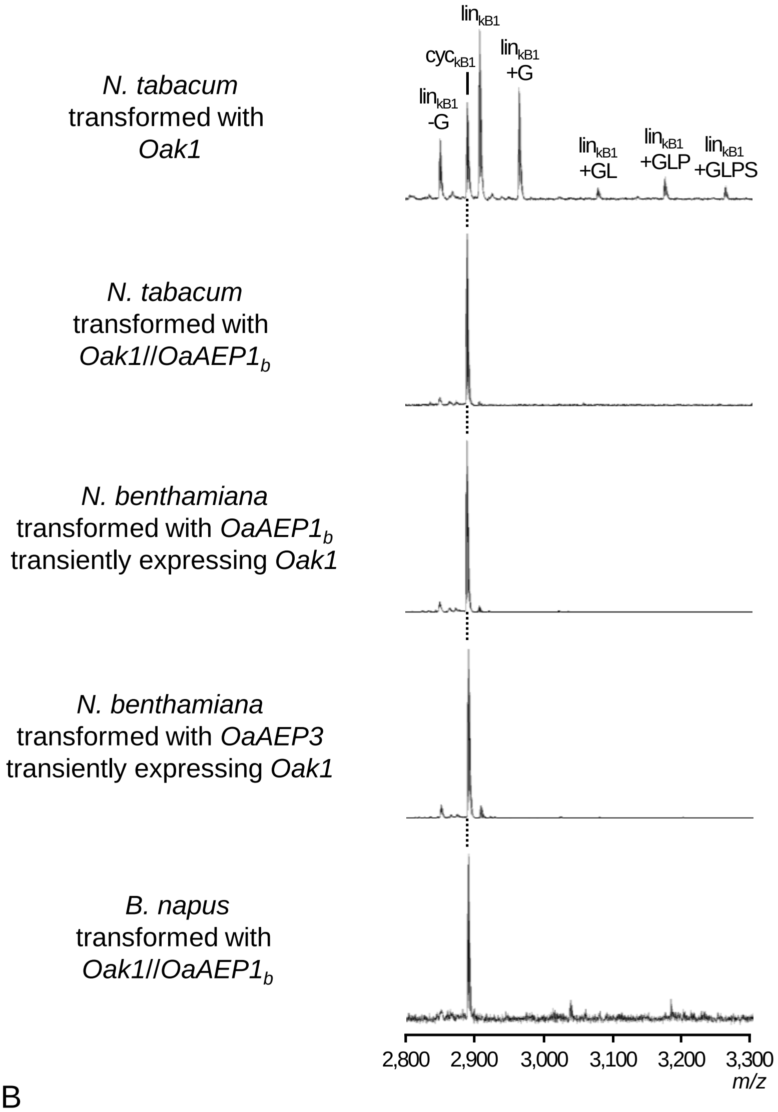

B

Plant	Transformation	No. of primary events	Percentage cyclic kB1 in primary transformants
N. tabacum	*Oak1* (stable)	16	0*, 15, 16, 17, 18, 18, 18, 18, 19, 20, 20, 20, 21, 22, 22, 26
N. tabacum	*Oak1*//*OaAEP1*$_b$ (stable)	13	0*, 0*, 0*, 8, 21, 79, 83, 84, 87, 87, 89, 90, 97
N. benthamiana	*OaAEP1*$_b$ (stable)<br>*Oak1* (transient)	8	21, 80, 82, 84, 86, 86, 87, 89
N. benthamiana	*OaAEP3* (stable)<br>*Oak1* (transient)	15	4, 11, 12, 32, 53, 54, 68, 70, 79, 80, 83, 84, 84, 86, 86
B. napus	*Oak1*//*OaAEP1*$_b$ (stable)	9	100% cyclic kB1 detected from three events; neither cyclic nor linear kB1 detected from six events

*Neither cyclic nor linear kB1 were detected in these lines.

Fig. S5. Cyclotide production in stable transformants. (A) Representative MALDI-TOF MS spectra of peptides produced in primary *N. tabacum*, *N. benthamiana* and canola (*B. napus)* transformants. The position of cyclic kB1 is indicated with a dotted line; cyc, cyclic product; lin, linear product. (B) Percentage of cyclic kalata B1 relative to all assigned peptides based on mass spectra peak areas produced in primary *N. tabacum*, *N. benthamiana* or canola (*B. napus*) transformants.

A

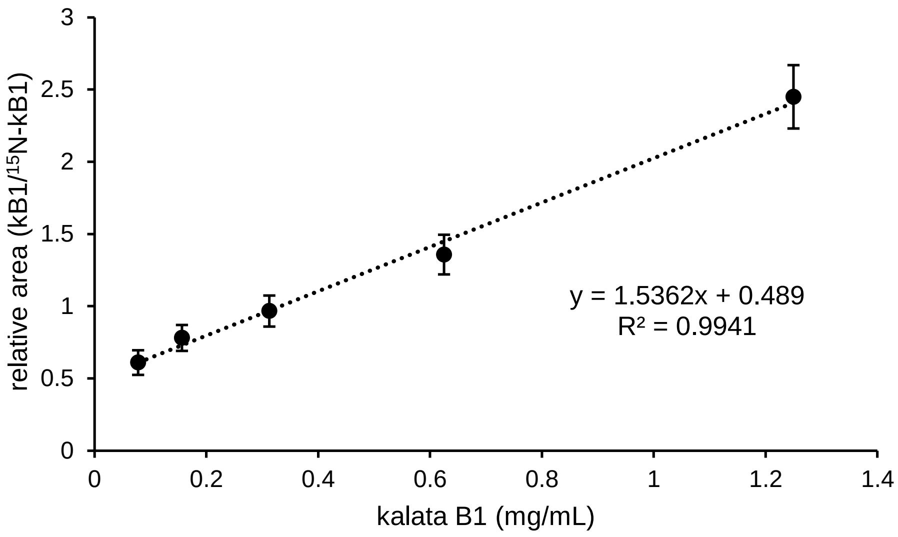

B

Transiently expressed construct	Cyclic kB1 concentration	
	mean μg g^{-1} DW	mean μg g^{-1} FW
pBIN19-*Oak1*	<8.5	<0.8
pBIN19-*Oak1* // *OaAEP1*$_b$	75.0 ± 7.3	6.9 ± 0.9
pBIN19-*Oak1* // *OaAEP1*$_b$ + pEAQexpress-*GFP-HT* (*p19*)	138.7 ± 12.8	20.6 ± 2.2
pEAQ-*HT-DEST1-Oak1*	<10.8	<1.7
pEAQ-*HT-DEST1-Oak1* (*N. benthamiana* line 7.5.5)	198.8 ± 20.7	29.4 ± 1.7

Fig. S6. MALDI-TOF MS quantitation of cyclic kalata B1 produced in *N. benthamiana*. (A) Standard curve for kB1 concentration using the relative MALDI-TOF MS peak area of kB1 to an ^{15}N-labelled kB1 internal standard. The equation and correlation co-efficient (R^2) are shown on the plot; each data point is the mean ± SEM (n=4). (B) The concentration of cyclic kB1 produced in *N. benthamiana* leaves expressed as μg g^{-1} dry weight (DW) or μg g^{-1} fresh weight (FW) ± SEM (n=3 biological replicates with each sample quantitated in triplicate). *N. benthamiana* line 7.5.5 is a single copy, homozygous line stably expressing pBIN19-*OaAEP1*$_b$; otherwise, wild-type *N. benthamiana* was used.

A

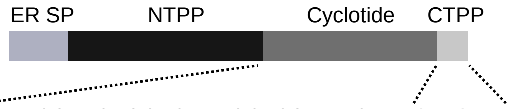

	Sequence	Relative % cyclic peptide reported previously when expressed alone
Oak1(WT)	...QLKGLPVCGETCVGGTCNTPGCTCSWPVCTRNGLPSLAA	5.1 ± 1.5%
Oak1(N29D)	...QLKGLPVCGETCVGGTCNTPGCTCSWPVCTRDGLPSLAA	0%
Oak1(N29A)	...QLKGLPVCGETCVGGTCNTPGCTCSWPVCTRAGLPSLAA	0%
Oak1(N29Q)	...QLKGLPVCGETCVGGTCNTPGCTCSWPVCTRQGLPSLAA	0%
Oak1(G30A)	...QLKGLPVCGETCVGGTCNTPGCTCSWPVCTRNALPSLAA	3.1 ± 1.7%
Oak1(L31A)	...QLKGLPVCGETCVGGTCNTPGCTCSWPVCTRNGAPSLAA	0%
Oak1(TRN*)	...QLKGLPVCGETCVGGTCNTPGCTCSWPVCTRN	0%
Oak1(G*)	...QLKGLPVCGETCVGGTCNTPGCTCSWPVCTRNG	0%
Oak1(GL*)	...QLKGLPVCGETCVGGTCNTPGCTCSWPVCTRNGL	3.7 ± 0.4%
Oak1(GLP*)	...QLKGLPVCGETCVGGTCNTPGCTCSWPVCTRNGLP	2.5 ± 1.9%

B

Oak1(N29D) + OaAEP1$_b$

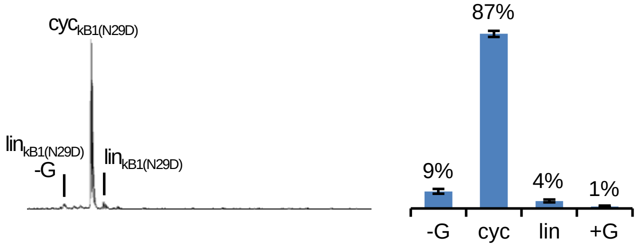

Observed mass	Assigned peptide
2853.4	linear kB1(N29D) - G
2892.5	cyclic kB1(N29D)
2910.5	linear kB1(N29D)/- G + G
2967.5	linear kB1(N29D) + G

Oak1(N29A) + OaAEP1$_b$

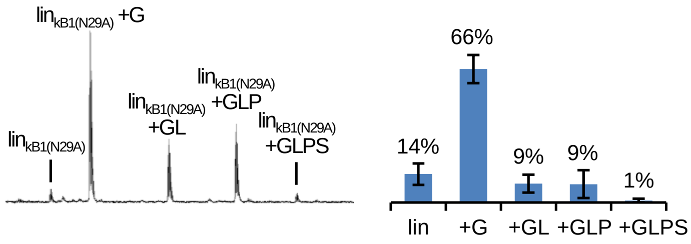

Observed mass	Assigned peptide
2866.6	linear kB1(N29A)/- G + G
2923.6	linear kB1(N29A) + G
3037.4	linear kB1(N29A) + GL
3134.2	linear kB1(N29A) + GLP
3220.5	linear kB1(N29A) + GLPS

Oak1(N29Q) + OaAEP1$_b$

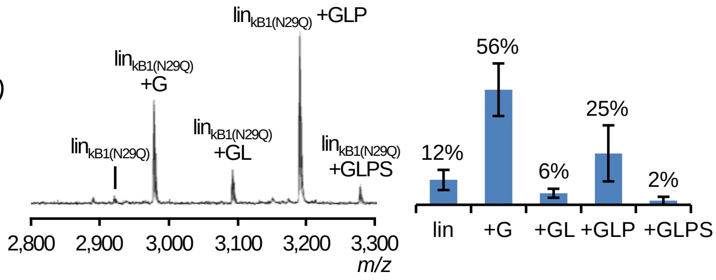

Observed mass	Assigned peptide
2923.7	linear kB1(N29Q)/- G + G
2980.7	linear kB1(N29Q) + G
3093.9	linear kB1(N29Q) + GL
3190.9	linear kB1(N29Q) + GLP
3277.9	linear kB1(N29Q) + GLPS

(b) (cont.)

Oak1(G30A) + *OaAEP1*$_b$

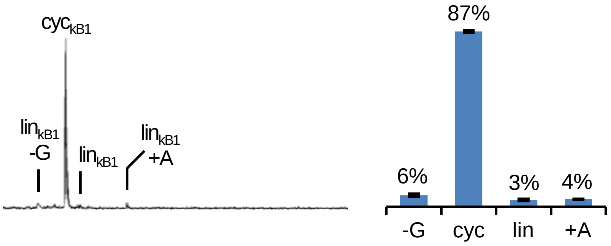

Observed mass	Assigned peptide
2852.7	linear kB1 - G
2891.8	cyclic kB1
2909.8	linear kB1
2980.9	linear kB1 + A

Oak1(L31A) + *OaAEP1*$_b$

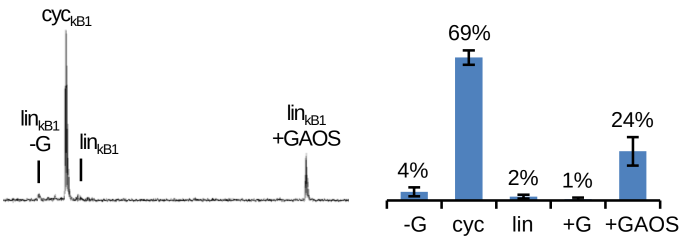

Observed mass	Assigned peptide
2852.7	linear kB1 - G
2891.7	cyclic kB1
2909.7	linear kB1/- G + G
2966.8	linear kB1 + G
3238.0	linear kB1 + GAOS

Oak1(TRN)* + *OaAEP1*$_b$

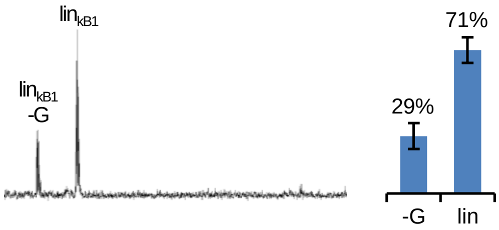

Observed mass	Assigned peptide
2852.6	linear kB1 - G
2909.6	linear kB1

Oak1(G)* + *OaAEP1*$_b$

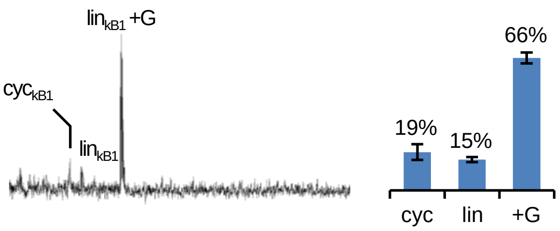

Observed mass	Assigned peptide
2892.0	cyclic kB1
2909.9	linear kB1/- G + G
2966.9	linear kB1 + G

Oak1(GL)* + *OaAEP1*$_b$

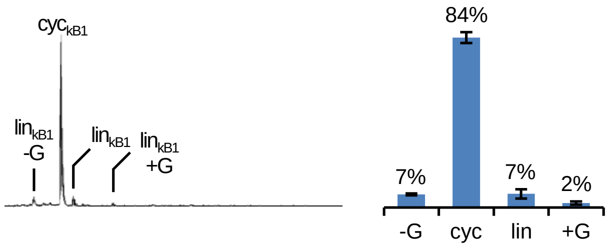

Observed mass	Assigned peptide
2852.7	linear kB1 - G
2891.7	cyclic kB1
2909.8	linear kB1/- G + G
2966.8	linear kB1 + G

Oak1(GLP)* + *OaAEP1*$_b$

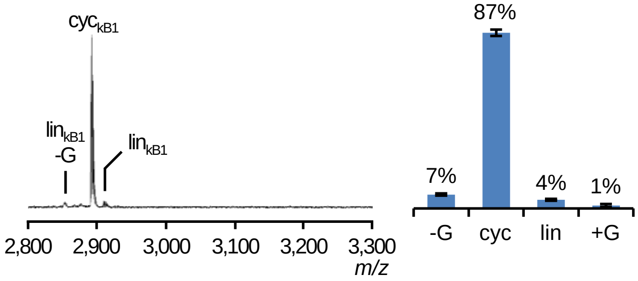

Observed mass	Assigned peptide
2852.7	linear kB1 - G
2891.7	cyclic kB1
2909.7	linear kB1/- G + G
2966.8	linear kB1 + G

C

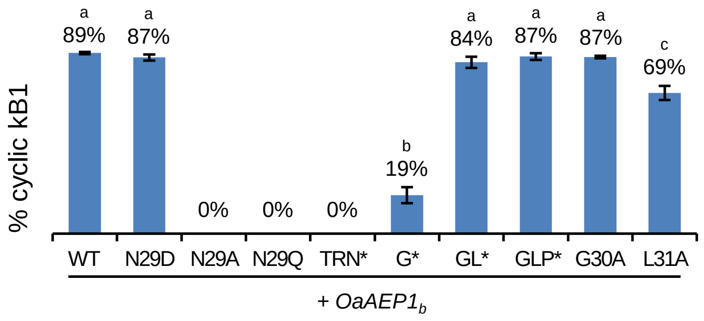

Fig. S7. Transient co-expression of *Oak1* variants with *OaAEP1*$_b$ in *N. benthamiana*. (A) Amino acid sequences of the cyclotide domain and CTPP of Oak1 wild type (WT) and variants and the relative percentages of cyclic peptide reported previously when expressed alone in either Arabidopsis, *N. tabacum* or *N. benthamiana* (Conlan *et al.,* 2012; Gillon *et al.,* 2008). (B) Representative MALDI-TOF MS spectra, the mean percentages of cyclic and linear products relative to all assigned peptides ± SEM based on mass spectra peak areas (n=3 except for *Oak1(N29D)* (n=6) and *Oak1(TRN*)* (n=5)) and the observed monoisotopic masses (Da; $[M+H]^+$) and assigned peptides; cyc, cyclic product; lin, linear product. (C) Comparison of the mean relative percentage of cyclic kB1 for each variant; different letters indicate significant differences found by Tukey's ANOVA ($p<0.05$).

A

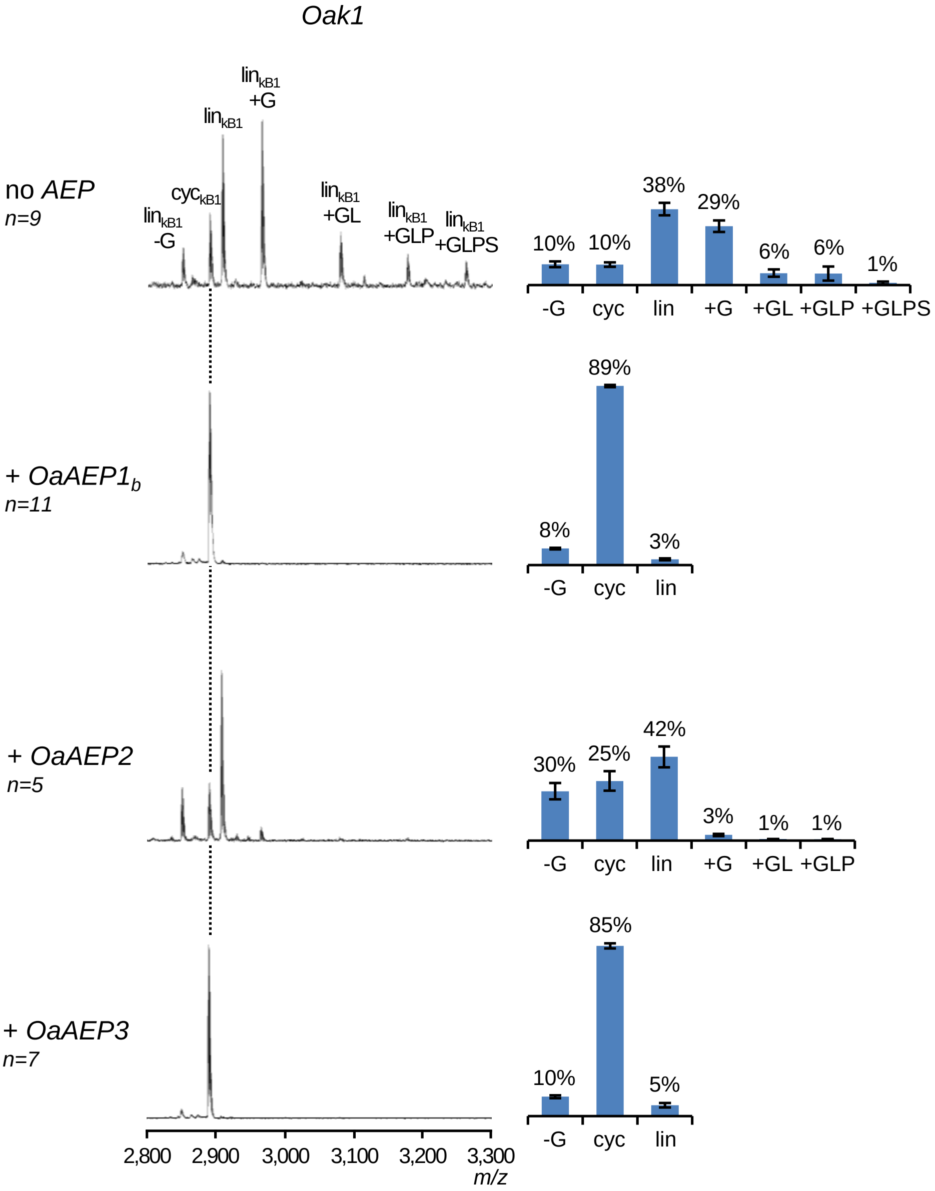

Observed mass	Assigned peptide
2852.4	linear kB1 - G
2891.4	cyclic kB1
2909.4	linear kB1/- G + G
2966.4	linear kB1 + G
3079.5	linear kB1 + GL
3176.6	linear kB1 + GLP
3262.9	linear kB1 + GLPS

B

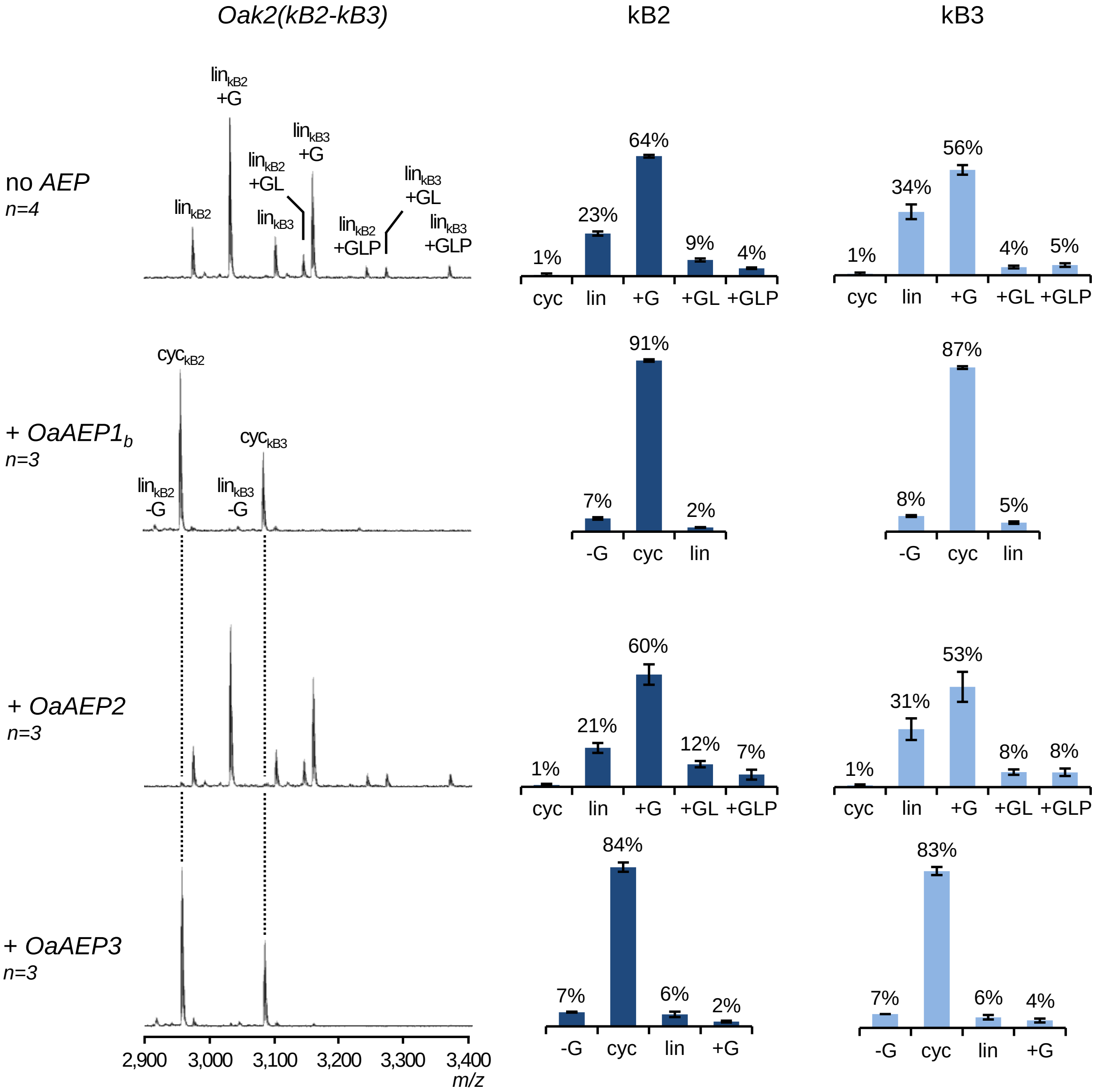

Observed mass	Assigned peptide
2915.3	linear kB2 - G
2954.3	cyclic kB2
2972.3	linear kB2/- G + G
3029.4	linear kB2 + G
3142.5	linear kB2 + GL
3239.5	linear kB2 + GLP

Observed mass	Assigned peptide
3042.3	linear kB3 - G
3081.3	cyclic kB3
3099.4	linear kB3/- G + G
3156.4	linear kB3 + G
3269.5	linear kB3 + GL
3366.6	linear kB3 + GLP

C

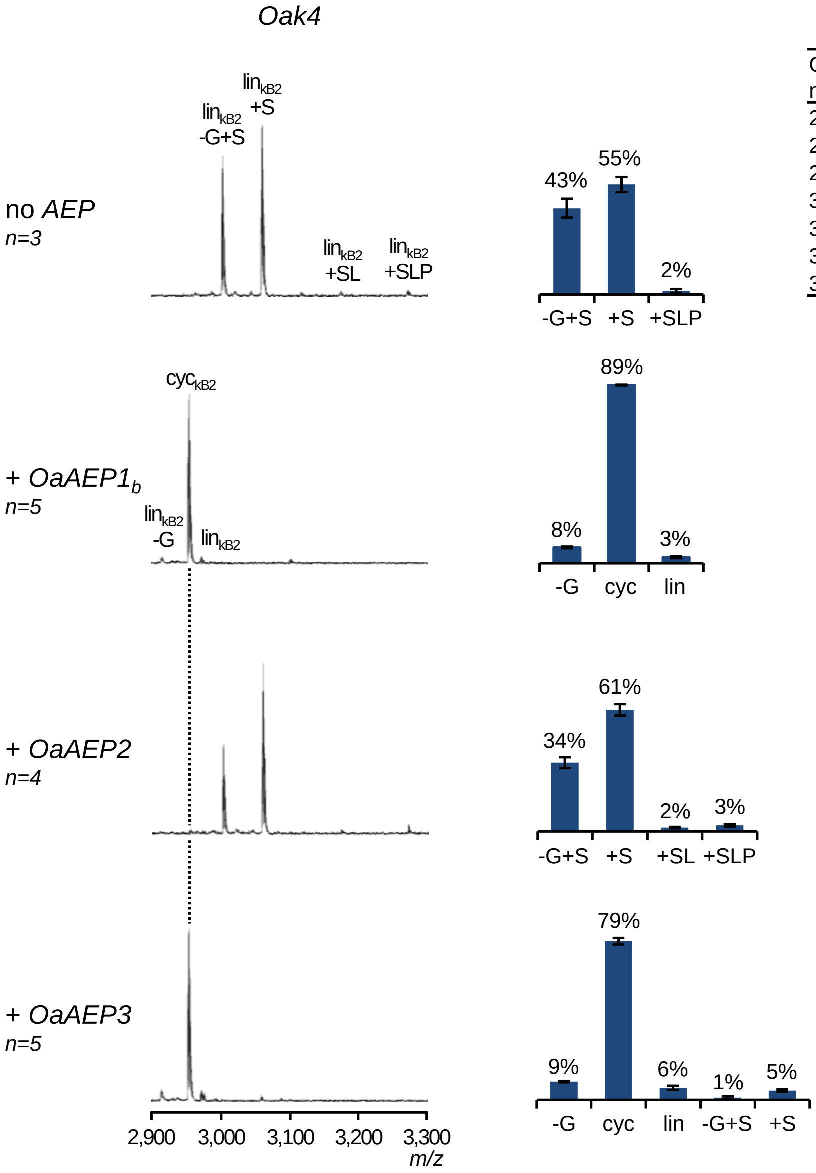

Observed mass	Assigned peptide
2915.2	linear kB2 - G
2954.3	cyclic kB2
2972.3	linear kB2
3002.2	linear kB2 - G + S
3059.3	linear kB2 + S
3172.2	linear kB2 + SL
3269.3	linear kB2 + SLP

D

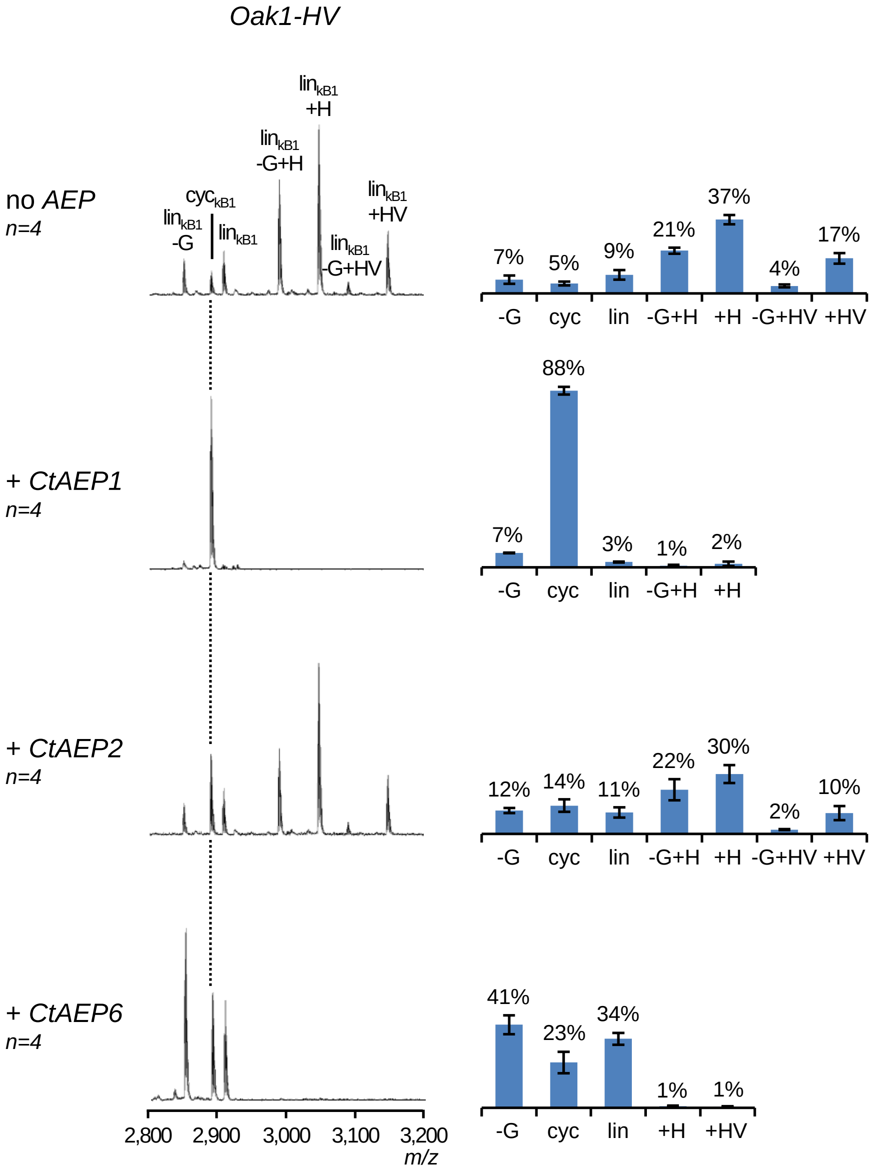

Observed mass	Assigned peptide
2852.2	linear kB1 - G
2891.3	cyclic kB1
2909.3	linear kB1
2989.3	linear kB1 - G + H
3046.3	linear kB1 + H
3088.4	linear kB1 - G + HV
3145.4	linear kB1 + HV

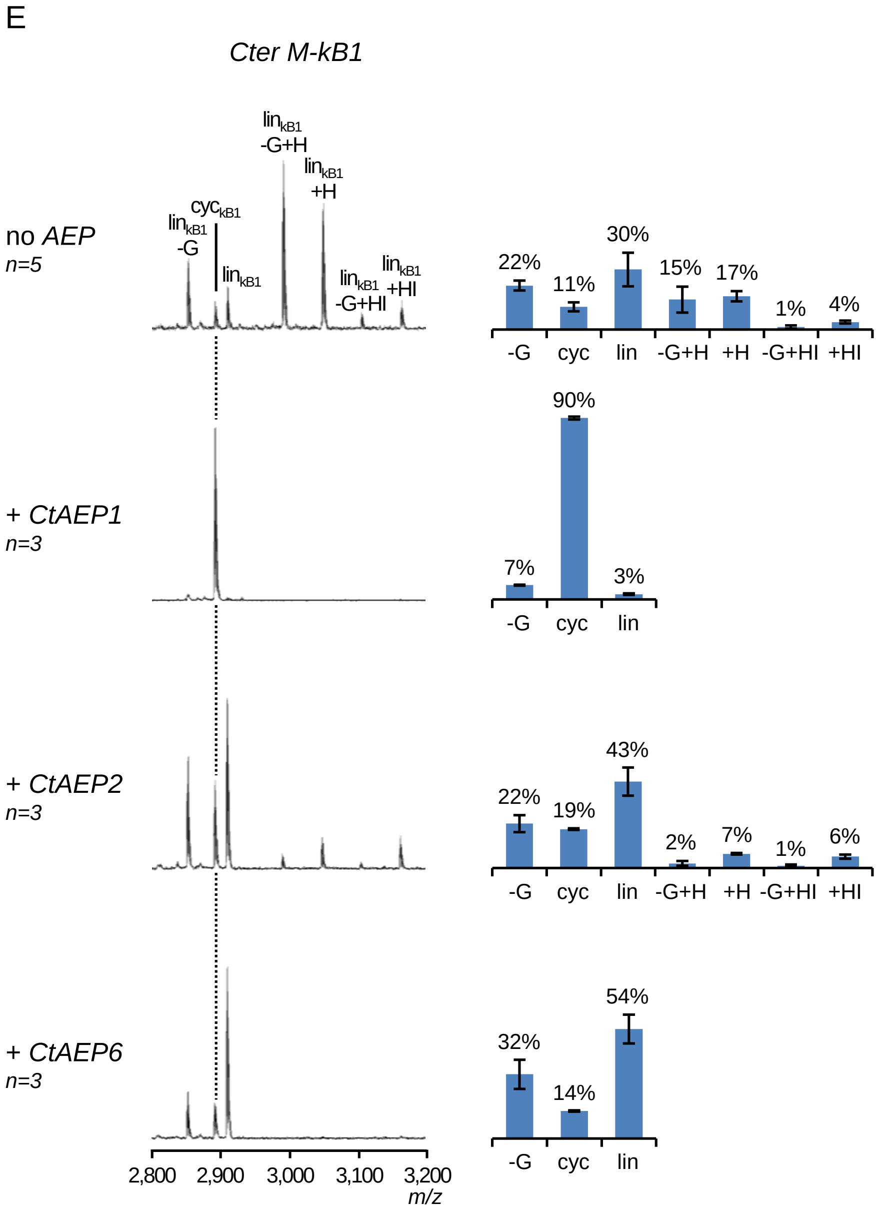

Observed mass	Assigned peptide
2852.6	linear kB1 - G
2891.6	cyclic kB1
2909.6	linear kB1
2989.7	linear kB1 - G + H
3046.7	linear kB1 + H
3102.6	linear kB1 - G + HI
3159.8	linear kB1 + HI

Fig. S8. Production of kalata B1, kalataB2 and kalata B3 by transient co-expression of the cyclotide precursors with a cyclizing AEP in *N. benthamiana*. Representative MALDI-TOF mass spectra of peptides produced by transient expression of (A) *Oak1*, (B) *Oak2(kB2-kB3)* and (C) *Oak4* alone or co-expressed with *OaAEP1$_b$*, *OaAEP2* or *OaAEP3* and (D) *Oak1-HV* and (E) *Cter M-kB1* alone or co-expressed with *CtAEP1, CtAEP2* or *CtAEP6.* The position of cyclic product is indicated with a dotted line; cyc, cyclic product; lin, linear product. The bar graphs show mean percentages of cyclic and linear products relative to all assigned peptides ± SEM based on mass spectra peak areas (n, number of independent replicates shown). The tables show observed monoisotopic masses (Da; $[M+H]^+$) and assigned peptides.

A

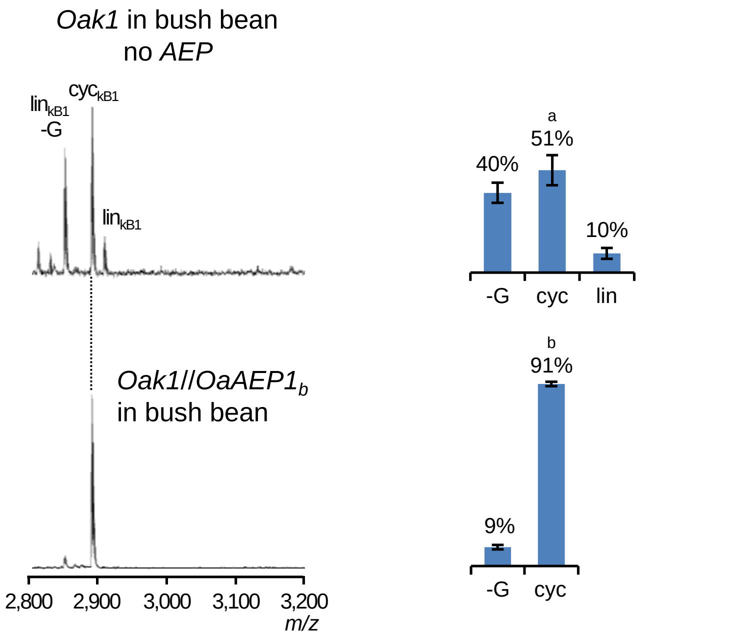

Observed mass	Assigned peptide
2852.4	linear kB1 - G
2891.4	cyclic kB1
2909.5	linear kB1/- G + G

B

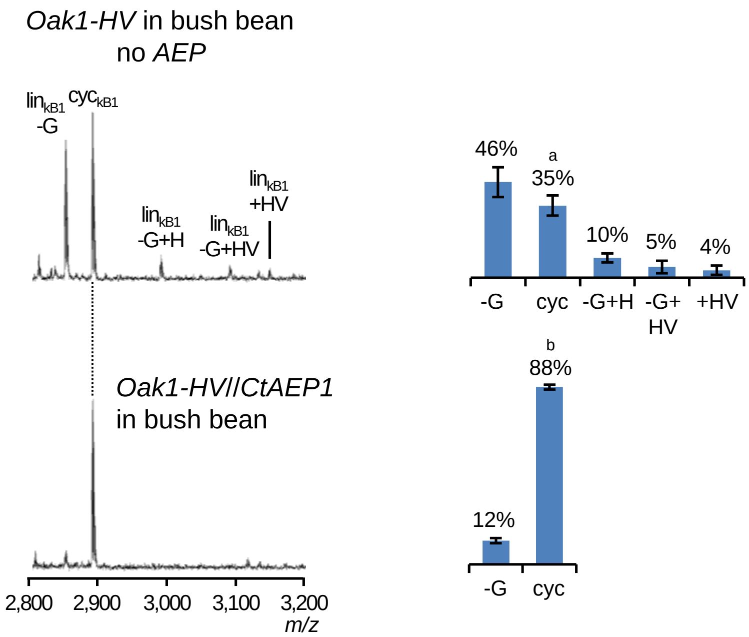

Observed mass	Assigned peptide
2852.4	linear kB1 - G
2891.4	cyclic kB1
2989.6	linear kB1 - G + H
3088.8	linear kB1 - G + HV
3145.7	linear kB1 + HV

(c)

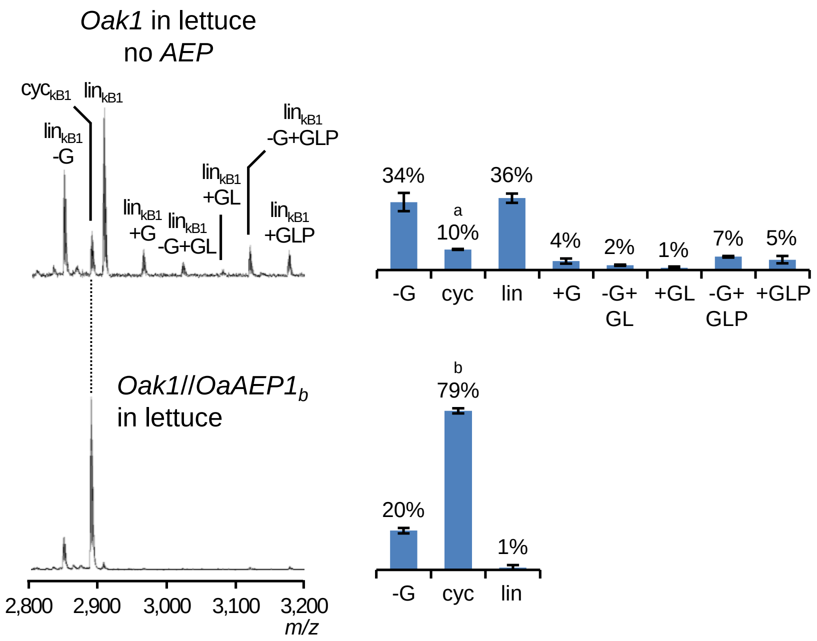

Observed mass	Assigned peptide
2852.2	linear kB1 - G
2891.3	cyclic kB1
2909.3	linear kB1/ - G + G
2966.6	linear kB1 + G
3022.4	linear kB1 - G + GL
3080.3	linear kB1 + GL
3119.4	linear kB1 - G + GLP
3176.5	linear kB1 + GLP

(d)

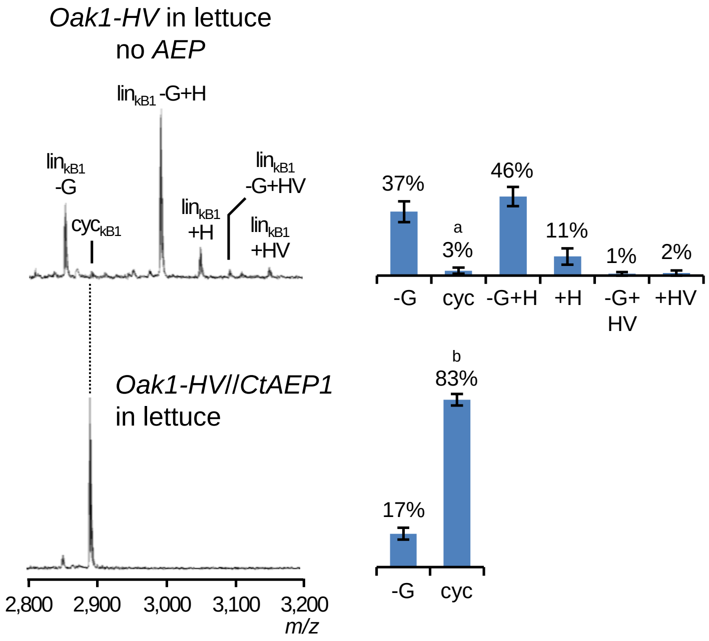

Observed mass	Assigned peptide
2852.3	linear kB1 - G
2891.3	cyclic kB1
2989.4	linear kB1 - G + H
3045.7	linear kB1 + H
3088.5	linear kB1 - G + HV
3145.5	linear kB1 + HV

Figure S9 Cyclotide production in bush bean and lettuce. Representative MALDI-TOF mass spectra of peptides produced by transient expression of (a, c) *Oak1* and (b, d) *Oak1-HV* either alone or in double stack constructs with *OaAEP1$_b$* or *CtAEP1*, respectively, in bush bean and lettuce. Mean percentages of cyclic and linear products relative to all assigned peptides ± SEM based on mass spectra peak areas (n=3). Different letters indicate significant differences found by Tukey's ANOVA ($p<0.05$); cyc, cyclic product; lin, linear product. The tables show observed monoisotopic masses (Da; $[M+H]^+$) and assigned peptides.

(a)

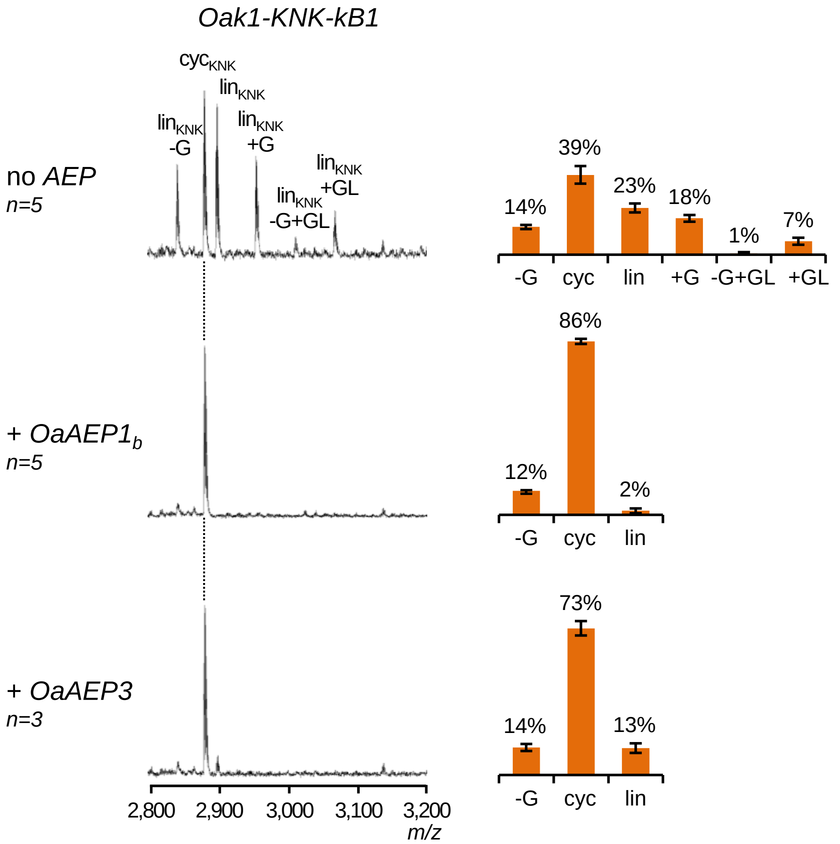

Observed mass	Assigned peptide
2840.1	linear KNK-kB1 - G
2879.2	cyclic KNK-kB1
2897.1	linear KNK-kB1/- G + G
2954.2	linear KNK-kB1 + G
3010.5	linear KNK-kB1 - G + GL
3067.4	linear KNK-kB1 + GL

(b)

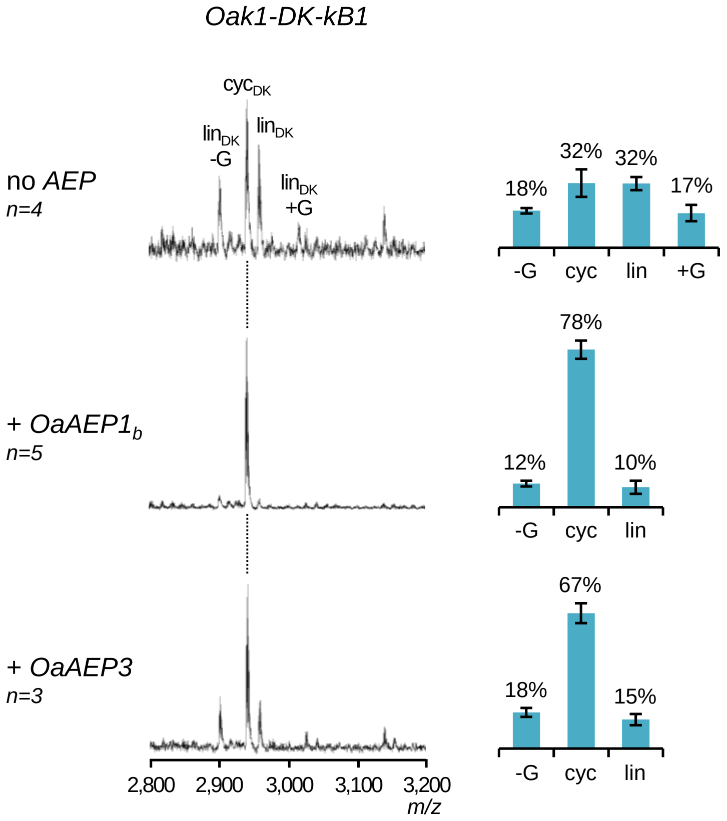

Observed mass	Assigned peptide
2899.0	linear DK-kB1 - G
2938.0	cyclic DK-kB1
2956.0	linear DK-kB1/- G + G
3013.0	linear DK-kB1 + G

C

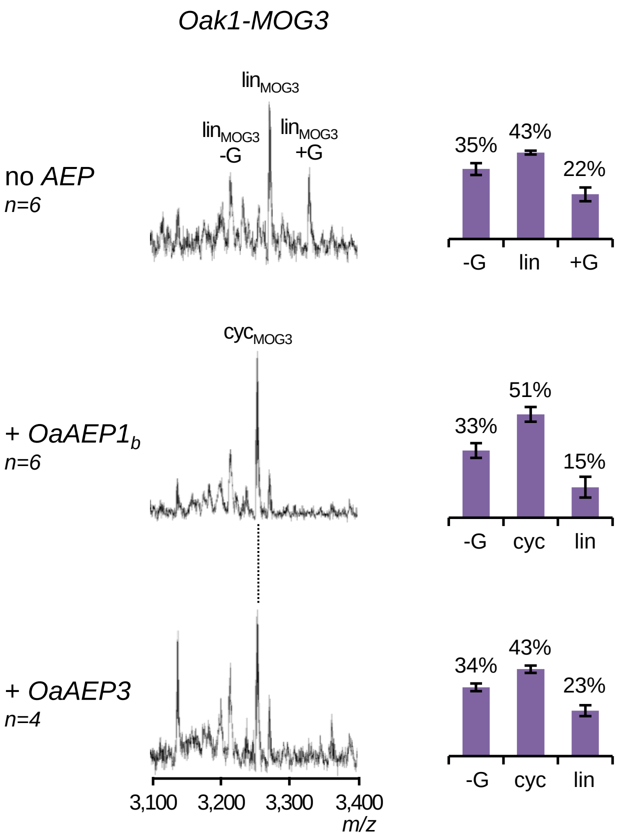

Observed mass	Assigned peptide
3212.2	linear MOG3 - G
3251.1	cyclic MOG3
3269.2	linear MOG3/- G + G
3326.1	linear MOG3 + G

D

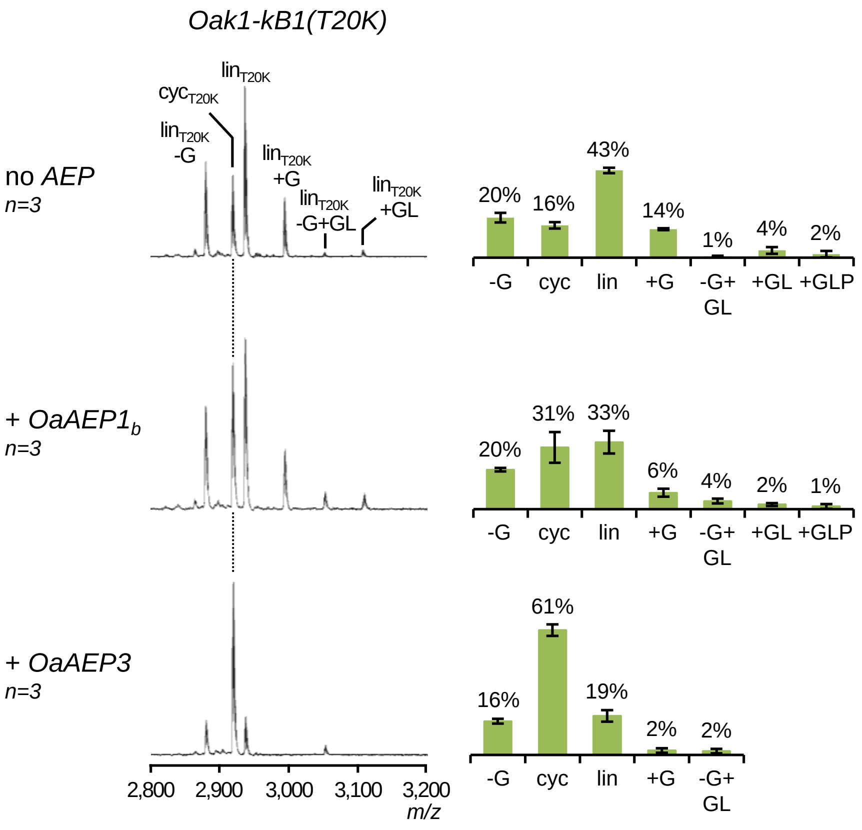

Observed mass	Assigned peptide
2879.3	linear kB1(T20K) - G
2918.3	cyclic kB1(T20K)
2936.3	linear kB1(T20K)/- G + G
2993.4	linear kB1(T20K) + G
3050.9	linear kB1(T20K) - G + GL
3106.4	linear kB1(T20K) + GL
3203.5	linear kB1(T20K) + GLP

E

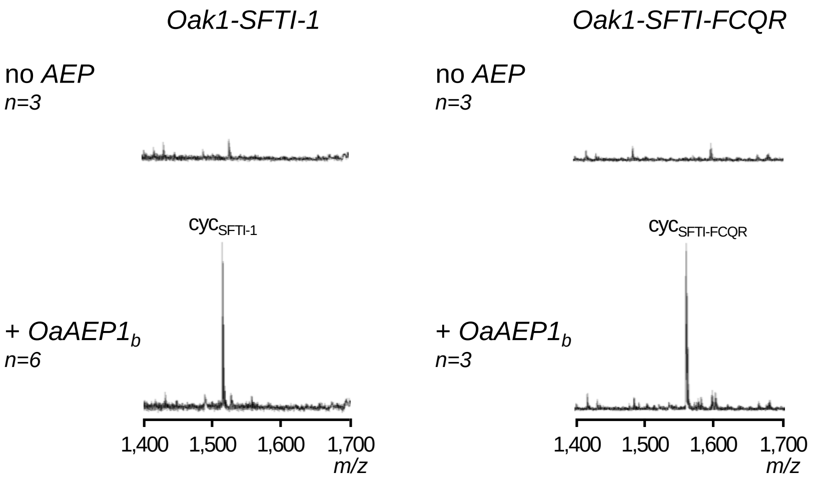

Observed mass	Assigned peptide
1513.7	cyclic SFTI-1
1559.6	cyclic SFTI-FCQR

F

```
KNK-kB1
ggacttccagtatgcggtgagacttgtgttgggggaacttgcaacactccaggctgcacttgctccaagaataagtgcacacgcaat
 G  L  P  V  C  G  E  T  C  V  G  G  T  C  N  T  P  G  C  T  C  S  K  N  K  C  T  R  N

DK-kB1
ggacttccagtatgcggtgagacttgtgttgggggaacttgcaacactccaggctgcacttgctcctgggataagtgcacacgcaat
 G  L  P  V  C  G  E  T  C  V  G  G  T  C  N  T  P  G  C  T  C  S  W  D  K  C  T  R  N

MOG3
ggacttccagtatgcggtgagacttgtgttgggggaacttgcaacactccaggctgcacttgcagatctccatttctagagtttgcacacgcaat
 G  L  P  V  C  G  E  T  C  V  G  G  T  C  N  T  P  G  C  T  C  R  S  P  F  S  R  V  C  T  R  N

kB1(T20K)
ggacttccagtatgcggtgagacttgtgttgggggaacttgcaacactccaggctgcaagtgctcctggcctgtttgcacacgcaat
 G  L  P  V  C  G  E  T  C  V  G  G  T  C  N  T  P  G  C  K  C  S  W  P  V  C  T  R  N

Oak1-SFTI-1 (codon-optimized for expression in N. tabacum)
atggctaagtttactgtgtgtcttttattgtgtttattattggctgcttttgttggtgcttttggttcagagttatcagattctcacaagactacactc
 M  A  K  F  T  V  C  L  L  L  C  L  L  L  A  A  F  V  G  A  F  G  S  E  L  S  D  S  H  K  T  T  L
gttaatgagattgctgagaagatgctccaaagaaaaatcttagatggagtggaagctacccttgttactgatgtggcagagaagatgtttttgaggaag
 V  N  E  I  A  E  K  M  L  Q  R  K  I  L  D  G  V  E  A  T  L  V  T  D  V  A  E  K  M  F  L  R  K
atgaaagctgaagcaaaaacatctgagaccgcagatcaggttttcttgaagcaacttcagttgaaaggtagatgcaccaagtctattcctcctatttgt
 M  K  A  E  A  K  T  S  E  T  A  D  Q  V  F  L  K  Q  L  Q  L  K  G  R  C  T  K  S  I  P  P  I  C
ttccctgatggattaccttctttagcagcataa
 F  P  D  G  L  P  S  L  A  A  -

Oak1-SFTI-FCQR: ttttgccagagg replaces agatgcaccaag in Oak1-SFTI-1
                 F  C  Q  R               R  C  T  K
```

Fig. S10. Production of grafted cyclic peptides in *N. benthamiana*. Representative MALDI-TOF mass spectra of peptides produced by transient expression of (A-D) the *Oak1* precursors for the grafted kalata B1 molecules, KNK-kB1, DK-kB1, MOG3 and kB1(T20K) either alone or co-expressed with *OaAEP1*$_b$ or *OaAEP3* and (E) the *Oak1* precursor where the kalata B1 cyclotide domain has been replaced with the mature SFTI-1 domain or the SFTI-1 domain containing the FCQR graft expressed alone or co-expressed with *OaAEP1*$_b$. The scales for the *Oak1-SFTI-1/SFTI-FCQR* ± *OaAEP1*$_b$ spectra are the same. The position of cyclic product is indicated with a dotted line; cyc, cyclic product; lin, linear product. The bar graphs show mean percentages of cyclic and linear products relative to all assigned peptides ± SEM based on mass spectra peak areas (n, number of independent replicates shown). The tables show observed monoisotopic masses (Da; $[M+H]^+$) and assigned peptides. (F) DNA and amino acid sequences of grafted cyclic peptides. The grafted kB1 sequences were expressed from within the *Oak1* precursor.
